# The Biochemical Properties of the Arabidopsis Ecto-Nucleoside Triphosphate Diphosphohydrolase AtAPY1 Contradict a Direct Role in Purinergic Signaling

**DOI:** 10.1371/journal.pone.0115832

**Published:** 2015-03-30

**Authors:** Carolin Massalski, Jeannine Bloch, Matthias Zebisch, Iris Steinebrunner

**Affiliations:** 1 Department of Biology, Technische Universität Dresden, Dresden, Germany; 2 Division of Structural Biology, University of Oxford, Oxford, United Kingdom; Leibniz-Institute for Vegetable and Ornamental Crops, GERMANY

## Abstract

The Arabidopsis E-NTPDase (ecto-nucleoside triphosphate diphosphohydrolase) AtAPY1 was previously shown to be involved in growth and development, pollen germination and stress responses. It was proposed to perform these functions through regulation of extracellular ATP signals. However, a GFP-tagged version was localized exclusively in the Golgi and did not hydrolyze ATP. In this study, AtAPY1 without the bulky GFP-tag was biochemically characterized with regard to its suggested role in purinergic signaling. Both the full-length protein and a soluble form without the transmembrane domain near the N-terminus were produced in HEK293 cells. Of the twelve nucleotide substrates tested, only three – GDP, IDP and UDP – were hydrolyzed, confirming that ATP was not a substrate of AtAPY1. In addition, the effects of pH, divalent metal ions, known E-NTPDase inhibitors and calmodulin on AtAPY1 activity were analyzed. AtAPY1-GFP extracted from transgenic Arabidopsis seedlings was included in the analyses. All three AtAPY1 versions exhibited very similar biochemical properties. Activity was detectable in a broad pH range, and Ca^2+^, Mg^2+^ and Mn^2+^ were the three most efficient cofactors. Of the inhibitors tested, vanadate was the most potent one. Surprisingly, sulfonamide-based inhibitors shown to inhibit other E-NTPDases and presumed to inhibit AtAPY1 as well were not effective. Calmodulin stimulated the activity of the GFP-tagless membranous and soluble AtAPY1 forms about five-fold, but did not alter their substrate specificities. The apparent K_m_ values obtained with AtAPY1-GFP indicate that AtAPY1 is primarily a GDPase. A putative three-dimensional structural model of the ecto-domain is presented, explaining the potent inhibitory potential of vanadate and predicting the binding mode of GDP. The found substrate specificity classifies AtAPY1 as a nucleoside diphosphatase typical of N-terminally anchored Golgi E-NTPDases and negates a direct function in purinergic signaling.

## Introduction

Ecto-nucleoside triphosphate diphosphohydrolases (E-NTPDases) break down nucleoside tri- and diphosphates (NTPs/NDPs) to nucleoside monophosphates (NMPs) and inorganic phosphate (P_i_) [1]. E-NTPDases can be stimulated by a variety of divalent ions. A more salient characteristic of E-NTPDases, however, is the presence of five conserved domains called “apyrase conserved regions” (ACRs) [2–4]. E-NTPDases occur predominantly in eukaryotes where they function extracellularly as well as within the cell. “Ecto” refers to the outside orientation of the catalytic domain facing the extracellular space or the lumen of an organelle [5]. It was proposed to reserve their historical name “apyrase” for intracellular E-NTPDases [5]. However, in the plant literature, the term “apyrases” is often used for extracellular E-NTPDases as well.

E-NTPDases perform a wide range of functions and therefore differ greatly in their enzymatic properties (reviewed in [4]). Extracellular E-NTPDases are considered to be involved in purinergic signaling in animals [6] and plants [7]. As regulators of purinergic signaling molecules such as ATP (adenosine triphosphate) and ADP (adenosine diphosphate), their catalytic efficiencies are reflected in low K_m_ values and high turnover numbers for these substrates [1]. The functions of intracellular E-NTPDases/apyrases vary for different localization sites. The lysosomal apyrase LALP70 is thought to facilitate the salvage of nucleotides [8], while the apyrases in the endoplasmic reticulum (ER) are believed to be involved in reglycosylation reactions [9,10] and the unfolded protein response [11]. Another proposed function is the control of the ATP concentration in the ER and Golgi lumen to regulate ATP-dependent processes [12]. A subset of Golgi apyrases, which do not hydrolyze ATP, but GDP (guanosine diphosphate) and UDP (uridine diphosphate), has functionally been characterized best. Deletion of their corresponding genes diminished the glycosylation of proteins in various yeast models [13–17] and in the nematode *Caenorhabditis elegans* [18]. The effect on glycosylation is based on the apyrase action of converting UDP and GDP to the corresponding NMP. This conversion is critical to sustain the activity of Golgi glycosyltransferases because of their inhibition by their by-products UDP and GDP [19].

In *Arabidopsis thaliana*, seven different candidate genes for E-NTPDases exist, but only four of the encoded proteins, AtAPY1 and 2 [20] and AtAPY6 and 7 [21], have been analyzed. AtAPY1 and 2 have a molecular weight of 51 kDa each and their hydropathy plots suggest that they are single-pass type II membrane proteins. Indeed, AtAPY1 was experimentally confirmed to be an integral membrane protein [22].

Their physiological roles have been studied in great detail. Overexpression of either *AtAPY1* or *AtAPY2* promoted growth as quantified in hypocotyl and pollen tube growth assays [23]. Reduced expression, on the other hand, e. g. by knocking out one of the two genes slowed down root hair growth compared with the wild type (WT) [24]. Knocking out both apyrase genes blocked pollen germination [25] and growth at the seedling stage [26]. Both blocks were abrogated by complementation with either *AtAPY1* or *AtAPY2* [25, 26], suggesting a high level of functional redundancy between the two very homologous proteins which share 87% sequence identity.

The model to explain the impact of AtAPY1 and 2 on pollen germination and growth was based on the idea that these processes were governed by extracellular ATP (eATP) signals [25, 27]. AtAPY1 and 2 would serve as the enzymes regulating the concentration of these signals, analogous to the situation established in animals. This idea was validated by the discovery of an ATP receptor in plants [28].

One of the key experiments that connected the growth promotions with AtAPY1 and 2 activities was an in-vitro pollen-tube-growth assay [23]. Adding polyclonal antibodies raised against AtAPY1 to growing pollen tubes inhibited extracellular soluble ATP hydrolysis activity and the growth rates of the pollen tubes. Simultaneously, the concentration of eATP rose.

The hypothesis of AtAPY1 and 2 limiting the concentration of eATP was further corroborated in studies on stomatal opening and closing [29]. All these findings also implied that the two apyrases were active outside of the cell.

In a direct approach to localize AtAPY1, it was tagged with green fluorescent protein (GFP) for detection in transgenic plants by confocal laser scanning microscopy and transmission electron microscopy. In contrast to the localization conclusions drawn from the pollen tube growth and stomata movement experiments, AtAPY1 was detected in the Golgi apparatus instead of the extracellular space [22]. The Golgi localization was confirmed independently by similar microscopy approaches using fluorescent-protein tagged AtAPY1 (and AtAPY2) [30, 31] and by identifying AtAPY1 in the Golgi proteome [31, 32].

Enzyme activity assays using isolated microsomes from mutant Arabidopsis lines indicated that AtAPY1-GFP and AtAPY2-GFP hydrolyze UDP and GDP, but not ADP or CDP [30]. A more comprehensive substrate specificity analysis with partially purified AtAPY1-GFP, testing twelve different nucleotides, revealed that only UDP, GDP and IDP (inosine diphosphate) served as substrates [22]. This substrate specificity suggested that AtAPY1 is more likely involved in glycosylation than in purinergic signaling.

In this study, AtAPY1 was analyzed in more detail, with a focus on its substrate specificity. Two GFP-tagless variants—one with and one without the transmembrane domain (TM)—were included to examine if the inability to break down ATP was caused by the fusion of AtAPY1 with the GFP-tag. Furthermore, the effect of calmodulin (CaM), which had been previously shown to bind to AtAPY1 [20], was investigated as a possible modifier of substrate specificity.

Our analysis provides a broad biochemical characterization of AtAPY1 and further evidence questioning its role in purinergic signaling.

## Materials and Methods

### Reagents

The nucleotide substrates, bovine calcineurin, NaF, NaN_3_ and Na_3_VO_4_ were purchased from Sigma and dissolved in water. Cellulase from *Trichoderma viride* was bought from Calbiochem. The inhibitor #1 (N-(3-methylphenyl)-4-biphenylsulfonamide) (see S1 Fig.) and inhibitor #4 (N’-(2-hydroxy-5-methylbenzylidene)-2-(1-naphthyl)acetohydrazide) (see S2 Fig.) were kindly provided by Gregory Clark and dissolved in dimethylformamide (DMF) at a final concentration of 5 mg/mL. Recombinant GFP (rGFP; 26.9 kDa) was purchased from ProSpec. The purified proteins hemagglutinin-enhanced GFP (HA-eGFP; 29.2 kDa), pea apyrase [33] and LpNTPDase1 (His_6_-tagged; [34]) were obtained from the group of Gerhard Rödel, Stanley Roux and Norbert Sträter, respectively.

### Plant culture

*Arabidopsis thaliana* wild-type (ecotype Wassilewskija) and transgenic *35S*:*AtAPY1-GFP* seedlings [22] were grown in liquid culture on a gyrotary shaker (100 rpm) in a 16-h light /8-h dark cycle or in continuous darkness. For each batch, 50 mg of surface-sterilized and overnight-stratified seeds were cultivated in a 250-mL Erlenmeyer flask containing 50 mL of culture medium (4.3 g/L Murashige and Skoog basal salt mixture, 0.5 g/L MES (2-(N-morpholino)ethanesulfonic acid), 1% (w/v) sucrose, pH 5.7 (adjusted with KOH)) supplemented with antibiotics (30 μg/mL kanamycin, 100 μg/mL ampicillin) for 10 to 12 d.

### Extraction, immobilization and quantitation of AtAPY1-GFP

First, a crude protein extract was prepared from drained seedlings as described elsewhere in detail [22]. Then, for the extraction of AtAPY1-GFP (for its sequence see S3 Fig.), 100–200 μL of protein extract (= 40–350 μg of protein depending on the yield) were added to microtiter plates coated with antibodies against GFP (GFP-multiTrap plates, ChromoTek, Planegg-Martinsried, Germany). After a minimum incubation of 2 h, the wells were washed three times with 300 μL of Tris-MES buffer (10 mM Tris, 2 mM MgCl_2_, 30 mM KCl, pH 6.5, adjusted with 1 M MES pH 3) for 2 min each at 500 rpm, so that only proteins immobilized by anti-GFP remained. The amount of bound protein was determined by measuring the GFP fluorescence. Each well to be analyzed was filled with 200 μL of Tris-MES buffer. The fluorescence (488 nm excitation/525 nm emission) was measured with the multimode plate reader TECAN infinite M200 (Tecan, Germany) at four different sites in each well (= “square filled 2 x 2” setting). The fluorescence was converted into protein amounts by using HA-eGFP as a reference. For the standard curve, the fluorescence of ten different amounts of HA-eGFP ranging from 0 to 810 ng of input protein per well was measured in duplicates.

### Cloning of AtAPY1 and AtAPY1-δTM

For production of C-terminally His-tagged AtAPY1 in HEK293 cells, the corresponding sequence *AtAPY1* (The Arabidopsis information resource: At3g04080) was cloned into the ApaI restriction site of the vector pcDNA3.1(-) (Life Technologies). The ApaI restriction sites, a Kozak consensus sequence and the sequence encoding the His-tag and the linker between AtAPY1 and its His-tag were added to the *AtAPY1* cDNA by PCR using the primers 5’-aagggcccattatgacggcgaagcgagcg-3’ and 5’-aagggccctcaatgatgatgatgatgatgtccacttcctggtgaggatactgcttctat-3’. For production of the C-terminally His-tagged soluble form of AtAPY1 in HEK293 cells, a shorter sequence encoding AtAPY1 without the TM was cloned into the AgeI and Acc65I restriction sites of the vector pHLsec. This vector contains sequences for a Kozak consensus, a secretion signal and a His_6_-tag [35]. The compatible restriction sites AgeI and BsiWI were introduced into the *AtAPY1* sequence by PCR using the primers 5’-ataaccggtccgaagaattacgctgtga-3’ and 5’-taacgtacgtgaggatactgcttctattg-3’. The encoded two AtAPY1 protein sequence versions are listed in S3 Fig.

### Transfection of HEK293 cells

HEK293 cells (1 x 10^8^) were resuspended in 50 mL of FreeStyle 293 expression medium (Life Technologies) without antibiotics. The expression plasmid (50 μg) was diluted in 5 mL of 150 mM NaCl containing 400 μg polyethylenimine (linear 25 kDa; Polysciences). The mixture was incubated for 10 min and then added to the cells. Four hours later, the cells were pelleted and resuspended in 100 mL of fresh FreeStyle 293 expression medium plus the antibiotics penicillin and streptomycin, and the culture incubation temperature was lowered from 37°C to 31°C. Cells were shaken at 100 rpm in an 8% CO_2_ in air atmosphere. Protein production levels were checked after 2, 3 and 4 d. For production analysis of AtAPY1, a culture sample of 1 x 10^6^ cells was taken each time point. The cells were pelleted, resuspended in 125 μL PBS containing 1 x Laemmli buffer and incubated at 80°C for 5 min. Nucleic acids were degraded with 20 U of benzonase at 37°C for 10 min. To check production levels of AtAPY1-δTM, 60 μL of cell culture supernatant per time point were mixed with Laemmli buffer to a final concentration of 1 x and incubated at 80°C for 5 min.

### Purification of His-tagged AtAPY1 and AtAPY1-δTM

Four days after transfection, the HEK293 cells were harvested. For purification of AtAPY1, about 1.5 x 10^8^ cells were resuspended in 2 mL of binding buffer (20 mM Tris, 500 mM NaCl, 10 mM imidazole, pH 7.5; 1 x EDTA-free protease inhibitor cocktail “cOmplete” (Roche)). After an ultrasonic treatment (75% power, 0.9 cycle, 5 x 1 s), 800 μL DNase I (1 mg/mL) were added, and the protein extract was incubated for 10 min on ice. Ni-NTA agarose (Qiagen) was equilibrated in binding buffer and incubated with the protein extract with an end-over-end rotation at 4°C for 1 h. The resin was pelleted, and a sample of the supernatant (= FT) was taken for further analysis. The pellet was washed with 1 mL of binding buffer (= W1). Three more washes (= W2–4) with 1 mL wash buffer each (20 mM Tris, 500 mM NaCl, 20 mM imidazole, pH 7.5; 1 x EDTA-free protease inhibitor cocktail “cOmplete” (Roche)) followed. Bound proteins were eluted with 1 mL elution buffer (20 mM Tris, 500 mM NaCl, 250 mM imidazole, pH 7.5) and dialyzed against Tris-MES buffer (10 mM Tris, 30 mM KCl, pH 6.5, adjusted with 1 M MES pH 3). The dialyzed proteins were diluted 1:1 in 100% (v/v) glycerol. The same purification procedure was followed for mock transfections, and the activity in the elution fraction was subtracted from the AtAPY1 activity values.

For purification of AtAPY1-δTM, the supernatant (40 mL) of a HEK293 culture 4 d post transfection was incubated with Ni-NTA resin (Qiagen) equilibrated in binding buffer (20 mM Tris, 500 mM NaCl, pH 8.0) with an end-over-end rotation at 4°C for 3 h. The culture supernatant (= S) contained 20% (v/v) glycerol and 1 x EDTA-free protease inhibitor cocktail “cOmplete” (Roche). The resin protein mix was transferred to a column and a sample of the flow through (= FT) was collected. Four washes (= W1–4) followed, each with 2 mL of binding buffer. Two more washes (= W5–6) ensued, each with 2 mL of wash buffer (20 mM Tris, 500 mM NaCl, 10 mM imidazole, pH 8.0). Bound proteins were eluted twice (= E1–2) with 0.5 mL of elution buffer each (20 mM Tris, 500 mM NaCl, 250 mM imidazole, pH 8.0). Dialysis and dilution in glycerol were performed as described above.

### Expression and purification of CaM

For purification of CaM, the *AtCaM2* cDNA (GenBank accession number AY065179) contained in the DNA clone U17248 (The Arabidopsis Information Resource database; [36]) was recombined into the pDEST17 vector (Life Technologies) by the Gateway technology (Life Technologies). This vector allowed N-terminal His_6_-tagging of AtCaM2. For gene expression, *E*. *coli* BL21 cells transformed with the vector were grown under antibiotic selection to an optical density) of 0.6 at 600 nm. Induction with 0.5 mM isopropyl-β-D-thiogalactopyranosid followed and cells were harvested 4 h later. For cell lysis, the pellet was resuspended in extraction buffer (20 mM Tris, 500 mM NaCl, pH 8.0; 1 x EDTA-free protease inhibitor cocktail “cOmplete” (Roche), 2 mM DTT, lysozyme (1 mg/mL)). After a 30-min incubation on ice, an ultrasonic treatment (75% power, 0.9 cycle, 6 x 10 s) was performed. The suspension was incubated with RNase A (1 mg/mL) on ice for 10 min. Cell debris was pelleted by centrifugation and the supernatant was filtered through a 0.22-μm mesh. The filtrate was adjusted with imidazole to a final concentration of 10 mM and subjected to Ni^2+^-affinity chromatography. Washes and elutions were performed with 20 mM and 250 mM imidazole, respectively. For NTPDase activity assays, the eluted protein was dialyzed against Tris-MES buffer (pH 6.5). For subsequent biotinylation, the dialysis was performed against 1 x PBS (pH 7.5).

### Biotinylation of CaM and SNAP-tag protein

For biotinylation of CaM, the EZ-Link Sulfo-NHS-SS-Biotin kit (Thermo Scientific) was used. CaM was concentrated via centrifugal concentration (5-kDa cut-off Vivaspin 500 concentrators; Sartorius) to a concentration of 6 mg/mL and then biotinylated according to the manufacturer’s instructions with a 20-fold molar excess of biotin reagent at 4°C overnight. Non-reacted Sulfo-NHS-SS-Biotin was removed by dialysis against 1 x PBS. Successful biotinylation was confirmed by dot or Western blotting and streptavidin-based detection described under “CaM overlay”.

The N-terminally His_12_-tagged SNAP-tag protein (New England Biolabs) was Ni^2+^-affinity purified and covalently labeled with biotin according to New England Biolabs’ instructions.

### Protein quantitation

Protein quantitation was performed in duplicates using the microplate procedure of a bicinchoninic acid (BCA) protein assay kit (Pierce).

### Silver and Coomassie staining

For staining of protein gels, the 1-step Coomassie staining solution from Generon was used or the silver staining procedure described in [37] was followed.

### Immunoblotting

Protein samples were separated by SDS-PAGE and transferred to BioTrace NT nitrocellulose or 0.45-μm PVDF membranes (both from PALL Life Sciences). The membranes were blocked in 1–5% (w/v) skim milk for 1 h and then incubated with primary antibodies for 1 h at room temperature or overnight at 4°C. The concentrations used were 1:1,000 for anti-GFP (mixture of two monoclonal mouse antibodies against recombinant GFP; Roche, catalog no. 11814460001), 1:50 for anti-AtAPY1 (polyclonal guinea pig antibody “gp19” against recombinant AtAPY1 [23], purified via CM Affi-Gel Blue (BioRad)) and 1:5,000 for anti-His (monoclonal mouse antibody against Penta-His; Qiagen, catalog no. 34660). Three washes for 10 min each followed before the horseradish peroxidase-coupled polyclonal secondary antibodies were added at the following concentrations: 1:5,000 for anti-mouse IgG from sheep (GE Healthcare, catalog no. NA9310V) and 1:10,000 for anti-guinea pig IgG from goat (Sigma, catalog no. A7289). After three more washes for 10 min each, the immunocomplexes were detected by chemiluminescence with the ECL Prime reagents (GE Healthcare). The primary and secondary antibodies were diluted in 1% (w/v) skim milk except for anti-His which was diluted in 3% (w/v) BSA. PBS- or TBS-based buffers were used for blocking, antibody dilutions and washes. Band intensities were quantified by densitometry using the TotalLab TL100 version 2006 software (Nonlinear Dynamics, UK).

### CaM overlay

For CaM overlays, PVDF membranes were used. All incubations were performed on a rocker at room temperature. After 15 min of preblocking (150 mM NaCl, 50 mM imidazole, pH 7.5), the membrane was blocked for 90 min (150 mM NaCl, 20 mM imidazole, 1 mM CaCl_2_, 1% (w/v) PVP-40, pH 7.5). The membrane was rinsed twice with wash buffer (150 mM NaCl, 20 mM imidazole, 1 mM CaCl_2_, 0.1% (w/v) PVP-40, 0.1% (v/v) Tween-20, pH 7.5) before it was incubated with AtCaM2 at a final concentration of 200 ng/mL in wash buffer for 2 h. Three washes with wash buffer for 7 min each followed. Streptavidin coupled to alkaline phosphatase (Vector Laboratories) was diluted 1:500 in wash buffer and added to the membrane. After 1 h, the streptavidin was washed off with three 7-min washes with wash buffer. Chemiluminescent signals were captured on film after a 5-min incubation in detection buffer (100 mM Tris, 150 mM NaCl, 1 mM CaCl_2_, Nitro-Block II (Tropix) diluted 1:20, CDP-Star (Roche) diluted 1:100, pH 9.5). As a control for the specificity of the CaM binding, another membrane was handled in parallel with the only difference that Ca^2+^ was replaced with 5 mM EGTA in all pertinent solutions.

### AtAPY1 activity assay

Apyrase activity was determined by the quantitation of P_i_ release per min by continuous and discontinuous methods. As controls, 5 mU of recombinant potato apyrase (New England Biolabs) were incubated in the presence of 3 mM ATP (positive control) or 3 mM AMP (negative control) in a total volume of 60 μL. The data analysis was performed with the Prism 5.0 software (GraphPad).

For the continuous AtAPY1 activity assay, the release of P_i_ was measured in the presence of 1 mM MgCl_2_ using the EnzChek phosphate assay kit (Molecular Probes). The kit is based on a coupled enzyme reaction described in [38]. Basically, the P_i_ produced by apyrase is the substrate of PNP (nucleoside phosphorylase) which converts P_i_ and 2-amino-6-mercapto-7-methylpurine riboside (MESG) to ribose 1-phosphate and 2-amino-6-mercapto-7-methylpurine (AMM). The product AMM absorbs light at 360 nm. Each standard reaction of 100 μL contained 0.1 U PNP and 200 μM MESG and was incubated at pH 6.5 and 25°C. One unit (U) PNP was defined as the phosphorolysis of 1.0 μmol of inosine to hypoxanthine and ribose 1-phosphate per minute at pH 7.4 and 25°C. The absorption was measured every 30 s with the multimode plate reader infinite M200 (Tecan, Germany).

The discontinuous activity assay was based on the method described in [39]. The nucleotide substrates were purchased from Sigma and the stock solutions were prepared in water. For the assays with AtAPY1-GFP, nucleotides were diluted in Tris-MES buffer (10 mM Tris base, 2 mM MgCl_2_ (unless stated otherwise in the figure legends), 30 mM KCl, pH 6.5 (except pH experiments), adjusted with 1 M MES pH 3) to the desired concentration and added as 130-μL aliquots to each well of immobilized AtAPY1-GFP on the GFP-multiTrap plate. The reaction was incubated under shaking (500 rpm) at 30°C for 1 h. The released phosphate was assayed by transferring 60 μL of each reaction mixture to two separate wells on a transparent 96-well microtiter plate (Cellstar, Greiner Bio-One, Kremsmünster, Austria) and by adding 120 μL of freshly prepared stopping solution of 0.375 M H_2_SO_4_, 0.75% (w/v) (NH_2_)_4_MoO_4_×4H_2_O, 0.7% (w/v) SDS and 3% (w/v) FeSO_4_×7H_2_O to each well. After a 10-min incubation at room temperature, the absorbance was read at 740 nm with the multimode plate reader infinite M200 (Tecan, Germany). A slightly modified version of the assay was performed for CaM and inhibitor #1 and #4 treatments. The nucleotides and either CaM or an inhibitor were diluted in the aforementioned Tris-MES buffer to the desired concentrations and added as 60-μL aliquots to each well of immobilized AtAPY1-GFP. For each treatment, two such reactions were run in parallel. After stopping the reactions by adding 120 μL of stopping solution to each well, the absorbance of the samples was measured directly in the GFP-multiTrap plate.

For the assays with AtAPY1 or AtAPY1-δTM, other modifications were made: The Tris-MES buffer used as reaction buffer contained 1 mM MgCl_2_, unless stated otherwise in the figure legends. Mastermixes including the enzymes and all necessary assay components except for the nucleotides were pipetted directly into the 96-well microtiter plates described above. The addition of nucleotides to initiate catalysis happened simultaneously. Reactions were run in duplicates and the final volume of each reaction was 60 μL. After the incubation period (for conditions see above), the reactions were terminated with 120 μL of stopping solution and assayed as outlined above. To determine the background from phosphate contaminations, unspecific phosphatase activities and non-enzymatic phosphate release during assaying, the following controls were set up: For the AtAPY1-GFP protein extracts, reactions were run in parallel with WT protein extracts of the same concentration. For the AtAPY1 and AtAPY1-δTM assays, control reactions were incubated at 95°C for 5 min to inactivate the enzymes before adding the substrates. The absorbance readings of the controls were subtracted from the sample readings.

### Structural modeling of the AtAPY1 ecto-domain

For selection of a template structure, sequence alignments of AtAPY1 (Universal protein resource knowledgebase (UniProtKB): Q9SQG2) and 2 (UniProtKB: Q9SPM5) to sequences of experimental NTPDase structures were performed. To date five different NTPDase structures have been solved [34, 40–45], all of which do not belong to the phylogenetic clade of intracellular NTPDases that also includes AtAPY1 and 2 [1]. Sequence identity of the catalytic domain of AtAPY1 to RnNTPDase1 (protein data bank (pdb): 3ZX3) is 22.6%, to RnNTPDase2 (pdb: 3CJA) 22.0% and to NTPDase1 from the bacterium *Legionella pneumophila* (LpNTPDase1; pdb: 3AAP) 22.5%. Sequence identities to the protozoan NTPDase of *Toxoplasma gondii* (pdb: 4A57) and *Neospora caninum* (pdb: 3AGR) are even lower. The RnNTPDase2 structure was selected over that of RnNTPDase1 and LpNTPDase1 for two reasons. First, all three cysteine bridges in the C-terminal domain are conserved between AtAPY1, 2 and RnNTPDase1, 2. LpNTPDase1, on the contrary, possesses only the two strictly conserved disulfide bridges. Second, multiple published and unpublished complex structures are available for RnNTPDase2 [45].

The sequence alignment between RnNTPDase2 and AtAPY1 performed with the ClustalW algorithm [46, 47] was manually cured in the nucleoside binding region to define equivalence of residues ^362^ASFFF^366^ of AtAPY1 to residues ^346^SAYYY^350^ of RnNTPDase2. Based on this alignment, a model for AtAPY1 containing residues K68 to P471 was generated using the program MODELLER [48]. For graphical reasons only, a single N-acetylglucosamine group was added manually in COOT [49] to the sole potential N-glycosylation site at N333. A model of a Mg^2+^×VO_4_^3-^ complex was obtained by copying the coordinates of the terminal phosphate and divalent metal cation of the Ca^2+^×AMPPNP (adenosine 5’-[(β,γ)-imido] triphosphate) complex of RnNTPDase2. Bond lengths of the artificial VO_4_^3-^ ion were corrected using COOT [49]. The validity of this approach is given because complex structures of RnNTPDase2 and LpNTPDase1 with phosphate, sulfate, tungstate, molybdate and vanadate could be obtained in which the anion always occupies the binding site of the terminal phosphate [34, 40, 45]. Molybdate and vanadate ions displayed distorted geometries reminiscent of a transition state, however, these distorted geometries have not been used in this work. A model of a Mg^2+^×GDP complex was obtained by copying the coordinates of the metal ion and the nucleotide analog of a Mg^2+^×GMPPNP (guanosine 5’-[(β,γ)-imido] triphosphate) complex of RnNTPDase2, but leaving out the coordinates of the α-phosphate moiety. As was published elsewhere [45], NTPDases were found to skip the first phosphate binding site in NDP hydrolysis.

### Statistical and bioinformatical analyses

Statistical analyses were performed with the programs Prism 5.0 (GraphPad) or SPSS 19 (IBM). TM and N-glycosylation site predictions were annotated according to the Knowledgebase [50] of the Universal Protein Resource [51]. Sequence alignments were done with the BioLign program version 4.0.6.2. The putative CaM binding sites were analyzed with an online analysis tool [52] described in [53]. Disulfide bond partner predictions were made with the help of the DiANNA 1.1 web server [54] described in [55].

## Results

### Bioinformatical analysis of the AtAPY1 protein sequence

**Fig 1:**
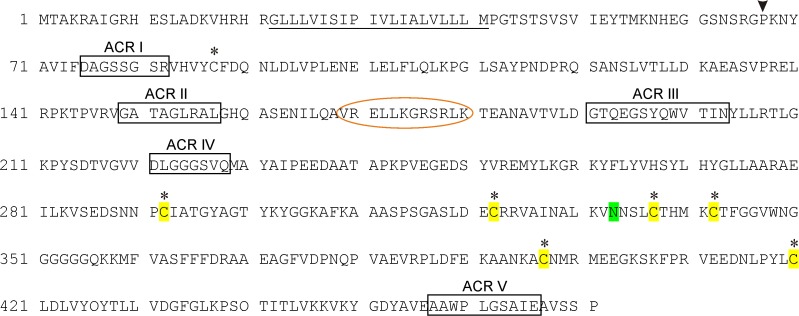
Protein sequence characteristics of AtAPY1. The complete AtAPY1 protein sequence is shown. The transmembrane region (TM) is underlined and the putative N-glycosylation site is marked in green. The ACRs are boxed. Six of the seven cysteines (black asterisks) are highly conserved and highlighted in yellow. The putative calmodulin-binding site is circled in orange. A black triangle marks where the sequence of AtAPY1-δTM begins.

The biochemical analysis of the protein sequence confirmed that AtAPY1 contains the five ACRs typical of E-NTPDases and a single TM near the N-terminus (Fig. 1). Furthermore, a N-glycosylation site and calmodulin-binding site (CBS) were found as published previously [20], although our analysis with the calmodulin target database [52] placed the most likely CBS (framed orange in Fig. 1) in a different position. Additionally, six highly conserved cysteines were identified which were predicted to form disulfide bridges.

Because of these predictions, extraction of AtAPY1 directly from its plant source was deemed important for biochemical analyses to ensure its native posttranslational modifications. In addition, when extracted from its native host, possible binding partners crucial for activity would get co-extracted.

Previously, a transgenic Arabidopsis line which synthesized AtAPY1 fused C-terminally with GFP was characterized [22]. The GFP-tag proved very useful in extracting active AtAPY1 and genetic complementation experiments gave no indication that the tag changed the biological function of AtAPY1 [22]. However, slight differences in enzyme properties between the tagged and the native AtAPY1 might have gone unnoticed. Therefore, AtAPY1 without the GFP-tag was included in our studies and expressed in the host HEK293 which harbors the potential of producing active proteins even if they contain TMs and require complex posttranslational modifications.

In addition, AtAPY1 without the TM was cloned and expressed in HEK293 cells to produce a soluble version, although there is no experimental evidence in the literature that a soluble form of AtAPY1 exists in vivo. This variant was included in the analyses nonetheless, because a soluble variant was described for mammalian homologs of AtAPY1 [56, 57].

**Fig 2:**
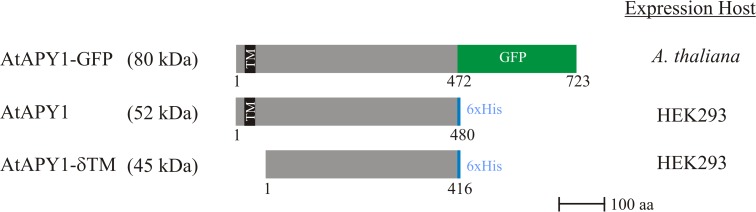
Scheme of the three AtAPY1 proteins analyzed. All three AtAPY1 protein versions analyzed are drawn to scale. The numbers refer to the first and last amino acid (aa) of each mature protein produced in either *A*. *thaliana* or human embryonic kidney cells (HEK) 293. For AtAPY1-GFP, the number 472 marks the first aa of the GFP-tag (in green). The other two AtAPY1 derivatives were tagged with six histidines (in blue). The theoretical molecular weight of the fusion proteins are given in parentheses. The transmembrane domain (TM) is depicted as a black bar. The absence of the TM in AtAPY1 is denoted by “-δTM”.

In total, three different versions of AtAPY1 as outlined in Fig. 2 were biochemically characterized.

### Isolation of AtAPY1-GFP

Active AtAPY1-GFP was extracted from transgenic Arabidopsis plants expressing *AtAPY1-GFP* in a one-step purification procedure using microtiter plates coated with antibodies against GFP (GFP-multiTrap plate). This technology was described to be a reproducible and robust isolation method of GFP fusion proteins [58]. In order to confirm this, equal volumes of the same crude protein extracts from *AtAPY1-GFP* expressing plants or WT control were added to wells of a GFP-multiTrap plate. After the incubation time, the protein solution was removed, replaced by buffer and the GFP fluorescence was determined. In the control wells, the fluorescence values were barely above the noise level of the detection method, indicating the absence of bound GFP (S4 Fig.). In the wells incubated with extracts from *AtAPY1-GFP* expressing plants, however, the fluorescence was significantly above the background (6–7 times above the limit of quantitation), demonstrating that GFP had bound (S4 Fig.). On average 330 ng of GFP from transgenic crude extracts were immobilized per well. This amount represents only an estimate because the standard curve for the fluorescence values was based on GFP and not AtAPY1-GFP as a reference.

There were no statistical differences in the amounts of bound GFP from the same protein extract (p < 0.001; one-way ANOVA (analysis of variance) test and Tukey test), confirming the reproducibility of this isolation method.

**Fig 3:**
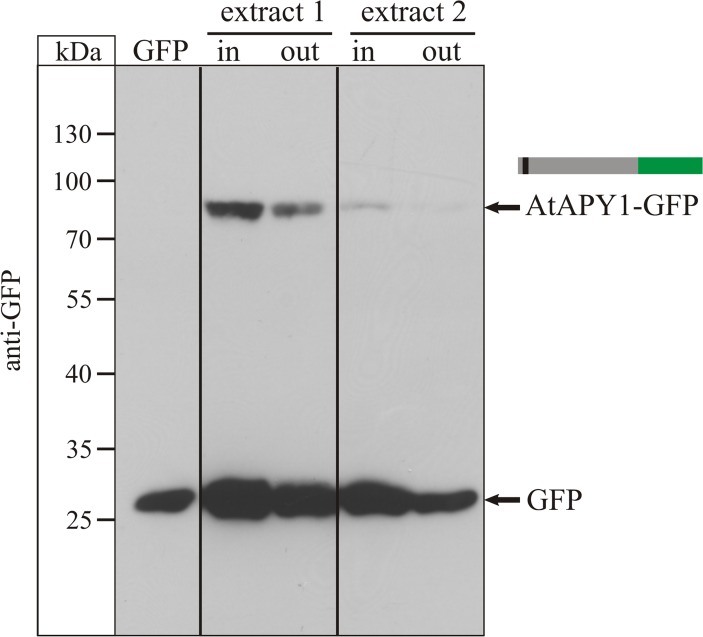
Detection of extracted AtAPY1-GFP. A Western blot analysis of two different crude protein extracts before (= in) and after (= out) the immobilization by GFP-multiTrap is shown. Equal volumes of extract (15 μL each) were loaded per lane, its proteins subjected to 10% SDS-PAGE, transferred to a nitrocellulose membrane and incubated with antibodies to GFP. The arrows mark the signals of the AtAPY1-GFP fusion protein and free GFP, respectively. The explanation of the colors in the schematic representation of AtAPY1-GFP can be found in Fig. 2. Recombinant GFP (19 ng) served as a positive control and quantitative reference for densitometric evaluation of signal intensities. With this, the total amounts of bound AtAPY1-GFP from 100 μL extract 1 and 2 were calculated as 130 ng and 22 ng, respectively. The image shows bands from the same exposure of the same membrane, but non-pertinent lanes were cropped as indicated by vertical lines. The shown signals are representative of at least five separate Western blot analyses of different GFP-multiTrap immobilization experiments.

In order to verify that AtAPY1-GFP was bound, the attempt was made to elute proteins bound by the GFP antibodies with 300 mM glycine (pH 2.5). However, the amount of proteins bound per well turned out to be too low to be detected by silver staining or even immunoblotting (data not shown). Therefore, an indirect approach was tested which turned out to be successful. The amount of AtAPY1-GFP in the crude protein extract before and after incubation in a GFP-multiTrap well was compared by Western blot analysis. As shown in Fig. 3, the antibody against GFP detected two proteins in the extracts: A protein of about 85 kDa which was close to the theoretical molecular weight of AtAPY1-GFP of 80 kDa, and a protein of around 27 kDa which matched the theoretical molecular weight of GFP alone. The bulk of free GFP most likely came from proteolytic degradation of AtAPY1-GFP during the protein extraction procedure and incubation period, although there is evidence from immunogold labeling experiments that degradation of AtAPY1-GFP also occurs in planta [22].

Comparing the signal intensities before and after immobilization showed that both free GFP and AtAPY1-GFP were captured by the GFP-multiTrap coating (Fig. 3). In addition, the immunoblotting results revealed that the majority of the GFP fluorescence originated from GFP cleaved off of AtAPY1 and that therefore the amount of bound AtAPY1-GFP could not be accurately determined by fluorescence measurements. The lack of precise numbers for bound AtAPY1-GFP amounts together with the unknown amount of unspecifically bound proteins, only allowed the calculation of the total activity for AtAPY1-GFP (Table 1). However, the GFP-multiTrap method proved to be a very sensitive method which allowed purifying active AtAPY1-GFP (as published in [22]) from little starting material reproducibly and fast.

**Table 1:** Characteristics of purified AtAPY1 proteins.

**Protein**	**Purification step**	**Total activity^a^ (nmol P_i_ /min)**	**Specific activity^a^ (μmol P_i_ /min x mg)**	**Fold purification**
AtAPY1-GFP	Binding to anti-GFP	0.09 + 0.03	n. d.	n. d.
AtAPY1	None^**b**^	0.37 + 0.39	0.78 + 0.95	n. a.
AtAPY1-δTM	None	2.04 + 0.43	0.33 + 0.09	n. a
	Ni^2+^-affinity chromatography	0.35 + 0.19	21.7 + 12.0	70

n. a., not applicable; n. d., not determinable

^a^Determined for the substrate UDP with the discontinuous assay. Values are the means from three assays + SD.

^b^Purification by Ni^2+^-affinity chromatography was not possible as described in Results.

### Expression and purification of AtAPY1 and AtAPY1-δTM

**Fig 4:**
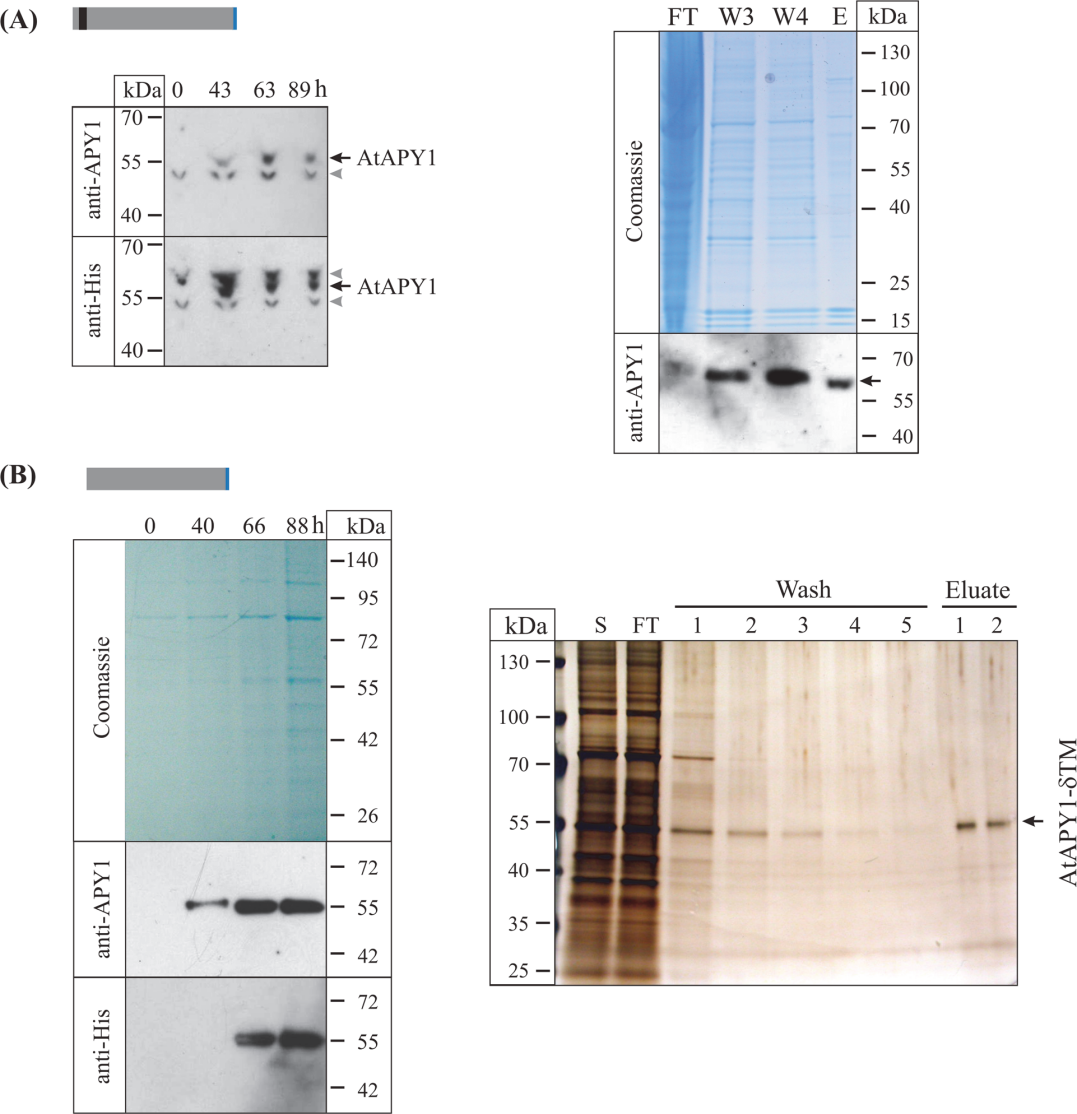
Purification of AtAPY1 and AtAPY1-δTM. The explanation of the colors in the schematic representations of AtAPY1 and AtAPY1-δTM can be found in Fig. 2. (A) Total proteins from 1.6 x 10^5^ HEK293 cells were harvested at each of the indicated time points post transfection with *AtAPY1* DNA, separated in a 4–12% gradient gel under denaturing conditions, transferred onto a PVDF membrane and successively incubated with anti-APY1 and anti-His antibodies (left panel). The black arrows mark the signal specific for AtAPY1, while the gray arrowheads indicate unspecific bands. The right panel shows the total protein extract from 1.4 x 10^8^ HEK293 cells harvested at 89 h after transfection with *AtAPY1* DNA subjected to Ni^2+^-affinity chromatography. Various fractions were separated in a 4–12% gel under denaturing conditions and either stained with Coomassie or transferred onto a PVDF membrane for Western blot analysis. The black arrow indicates the signal detected with antibodies against AtAPY1. The volumes loaded were 1/480 of the flow through (FT) fraction, 1/50 of each of the final two wash fractions W3 and W4 and 1/100 of the elution fraction E. (B) The left panel shows samples representing equal volumes (1/3,000) of the culture medium of 1 x 10^8^ HEK293 cells taken at the indicated time points post transfection with *AtAPY1-δTM* DNA and separated in a 4–12% gradient gel under denaturing conditions. Subsequently, the proteins were either stained with Coomassie or blotted onto a PVDF membrane for Western blot analysis. The right panel depicts the culture medium of 4 x 10^7^ HEK293 cells at time point 88 h after transfection with *AtAPY1-δTM* DNA subjected to Ni^2+^-affinity chromatography. A gradient gel (4–12%) was loaded with 20 μL of supernatant (S) and 20 μL of flow through (FT), 10 μL of each wash 1–5 and 10 μL of each eluate 1–2. For total volumes of the individual fractions see Materials and Methods. The protein amount loaded for eluate 1 equals about 70 ng. Following SDS-PAGE, the gel was silver-stained.

For the production of a GFP-tagless version, the cloned AtAPY1 sequence was transfected into HEK293 cells. At various time points after transfection, cell extracts were prepared and subjected to Western blot analysis. Using an antibody against AtAPY1, two proteins were detected (Fig. 4A, left panel). The smaller protein of 52 kDa was regarded as unspecific, because it was already present before expression of *AtAPY1* occurred. The larger protein of 61 kDa, however, was absent at time point zero and appeared only at later time points, making it a likely candidate for AtAPY1. Since AtAPY1 was tagged C-terminally with six histidines, it was tested if an antibody against the His-tag would detect the same 61-kDa protein (Fig. 4A, left panel). Indeed, the His antibody recognized the protein in question. Two other proteins of 65 kDa and 52 kDa were recognized as well, but they were disregarded due to their presence at time point zero. The recognition of the 61-kDa protein strengthened the argument that this protein represented AtAPY1, although the apparent molecular weight was higher than the theoretical molecular weight of 52 kDa.

In summary, the Western blot analyses suggested that AtAPY1 was synthesized by the HEK293 cells and that the amount of AtAPY1 remained approximately the same over four days post transfection.

Since the His-tag was shown to be present, Ni^2+^-affinity purification of AtAPY1 was attempted. However, Coomassie staining of various purification fractions showed no sign of enrichment of AtAPY1 in the elution fraction (Fig. 4A, right panel). A Western blot analysis of the fractions with AtAPY1 antibodies confirmed that the amount of AtAPY1 remained similar in all fractions (Fig. 4A, right panel), as if AtAPY1 was not binding to the matrix. Therefore, less stringent binding and washing conditions with 5 mM imidazole instead of 20 mM were tried, but no binding was observed. Similarly, the addition of a detergent (20 mM 3-[(3cholamidopropyl)dimethylammonio]-1-propanesulfonate) and a reducing agent (2 mM dithiothreitol) to expose the possibly hidden His-tag did not promote binding.

When NTPDase activities were assayed, no activity was found in the elution (and other) fraction(s) from the purification steps using the cell extract from the mock transfected cells. The elution fraction from the transfected HEK293 cells, however, showed a specific activity of 0.78 + 0.95 U/mg (Table 1). So, although the specific activity was low, active AtAPY1 was being produced.

For the expression of the soluble form, the sequence encoding AtAPY1 from the first ß-strand after the TM to the end except for the terminal proline (residues 67–470) was cloned (Fig. 1). Sequences coding for a secretion signal peptide and a C-terminal His_6_-tag were added (Fig. 2, S3 Fig.). HEK293 cells transfected with this construct showed no production of a protein of the expected molecular weight of 45 kDa as judged by Coomassie staining of culture supernatants from day 2 to 4 after transfection (Fig. 4B, left panel). However, Western blots with antibodies against AtAPY1 unraveled the synthesis of a protein of 55 kDa (Fig. 4B, left panel). Even though the apparent molecular weight of this protein was higher than the theoretical molecular weight of AtAPY1-δTM, it was concluded that it represented AtAPY1-δTM because of its absence before transfection and its increasing amount over time. Incubation of the stripped membrane with His antibodies supported this conclusion as the same protein of 55 kDa was detected (Fig. 4B, left panel). The results with anti-His also confirmed the presence of the His-tag.

Since the amount of AtAPY1-δTM was highest four days after transfection (Fig. 4B, left panel), the culture supernatant from this time point was used for Ni^2+^-affinity chromatography (Fig. 4B, right panel). Silver staining of the fractions demonstrated that the purification was successful since a protein of 55 kDa was enriched in the elution fractions. Western blot analyses of several of these fractions with antibodies against the His-tag or AtAPY1 confirmed the identity of this 55-kDa protein as AtAPY1-δTM (S5 Fig.). NTPDase activity measurements demonstrated that AtAPY1-δTM was active. The increase of about 70 times between the specific activity of AtAPY1-δTM in the supernatant and the elution fraction (Table 1) validated the conclusion of a successful purification.

### Broad pH range for activity

**Fig 5:**
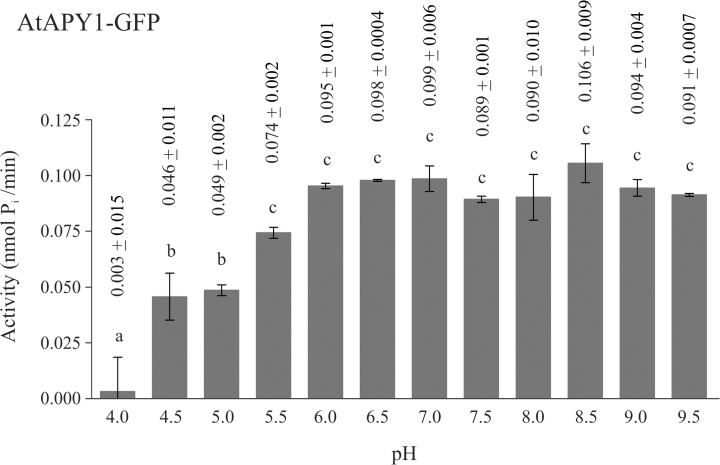
Influence of pH on AtAPY1-GFP activity. Enzyme activities were determined in the presence of 3 mM UDP using the discontinuous apyrase activity assay. The activity of AtAPY1-GFP was measured discontinuously over the pH range indicated. Different letters above the columns indicate mean values that are significantly different from one other (one-way ANOVA and Tukey test; p < 0.01). Error bars represent standard deviations of duplicates from one assay. The data are representative of four activity assays with independent protein extracts.

AtAPY1 was previously localized to the Golgi apparatus [22, 30–32]. Therefore, the pH optimum of AtAPY1 was expected to be around 6.2 which represents the pH found in the Golgi apparatus [59]. In order to test this assumption, the activity of AtAPY1-GFP was assayed in the pH range from 4.0 to 9.5 in 0.5 pH intervals. AtAPY1-GFP was particularly useful for this assay, because its binding to the plate well bottoms allowed the original liquid to be readily replaced by the reaction buffer of the desired pH. AtAPY1-GFP showed the highest and same level of activity from pH 5.5 to 9.5 (Fig. 5). There was a significant drop in activity (p < 0.01) at pH 5.0 and the activity decreased further with increasing acidity. At pH 4.0 the activity was down to 3% of the highest activity observed. In summary, AtAPY1-GFP showed maximum activity in a broad pH range which included the pH of its Golgi localization.

### Effect of divalent ions on activity

**Fig 6:**
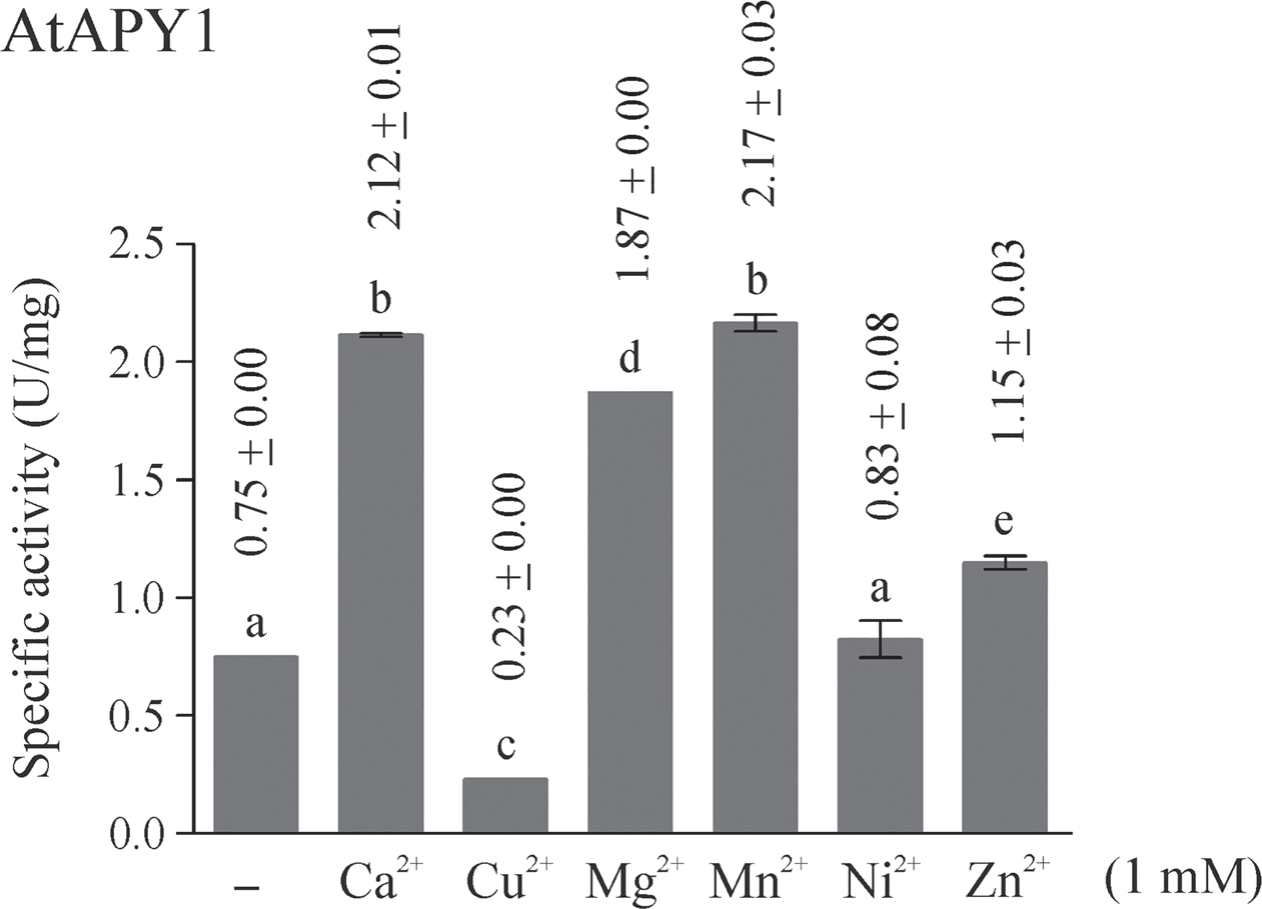
Influence of divalent metal ions on AtAPY1 activity. Enzyme activities were determined in the presence of 3 mM UDP using the discontinuous apyrase activity assay. The activity of AtAPY1 (1 U = 1 μmol P_i_ /min) was measured in the absence or presence of either 1 mM CaCl_2_, CuCl_2_, MgCl_2_, MnCl_2_, NiCl_2_ or ZnCl_2_. The control (-) shows the activity without the addition of any divalent ions. The means + SD of duplicates from one assay are shown. Different letters above the columns indicate mean values that are significantly different from one other (one-way ANOVA and Tukey test; p < 0.01). Data are representative of two activity assays.

E-NTPDases are known to depend on divalent ions for activity, because the metal nucleotide complex acts as the real substrate. Nevertheless, the most suitable metal as cofactor differs among NTPDases. For AtAPY1, six different divalent ions were tested (Fig. 6). The ions Ca^2+^, Mg^2+^, Mn^2+^ and to a lesser extent Zn^2+^ stimulated its activity while Ni^2+^ had no effect. The metal Cu^2+^ even inhibited its activity.

This metal ion preference was compared with that of AtAPY1-GFP (S6 Fig.). Both enzymes showed the same response pattern, except for AtAPY1-GFP not being stimulated by Zn^2+^. Since some activity was observed even in the absence of any cofactor (Fig. 6, S6 Fig.), it was checked if the residual activity was due to contaminating ions in the reaction buffer. For this control, the activity of AtAPY1-GFP with and without the chelator EDTA was compared (S6 Fig.). Indeed, the addition of EDTA completely abolished apyrase activity, pointing to the presence of some divalent ions in the reaction buffer. Therefore, as anticipated, the activity of AtAPY1 depends on divalent ions as cofactors which are preferably Ca^2+^, Mg^2+^ or Mn^2+^.

### Inhibitors of AtAPY1

**Fig 7:**
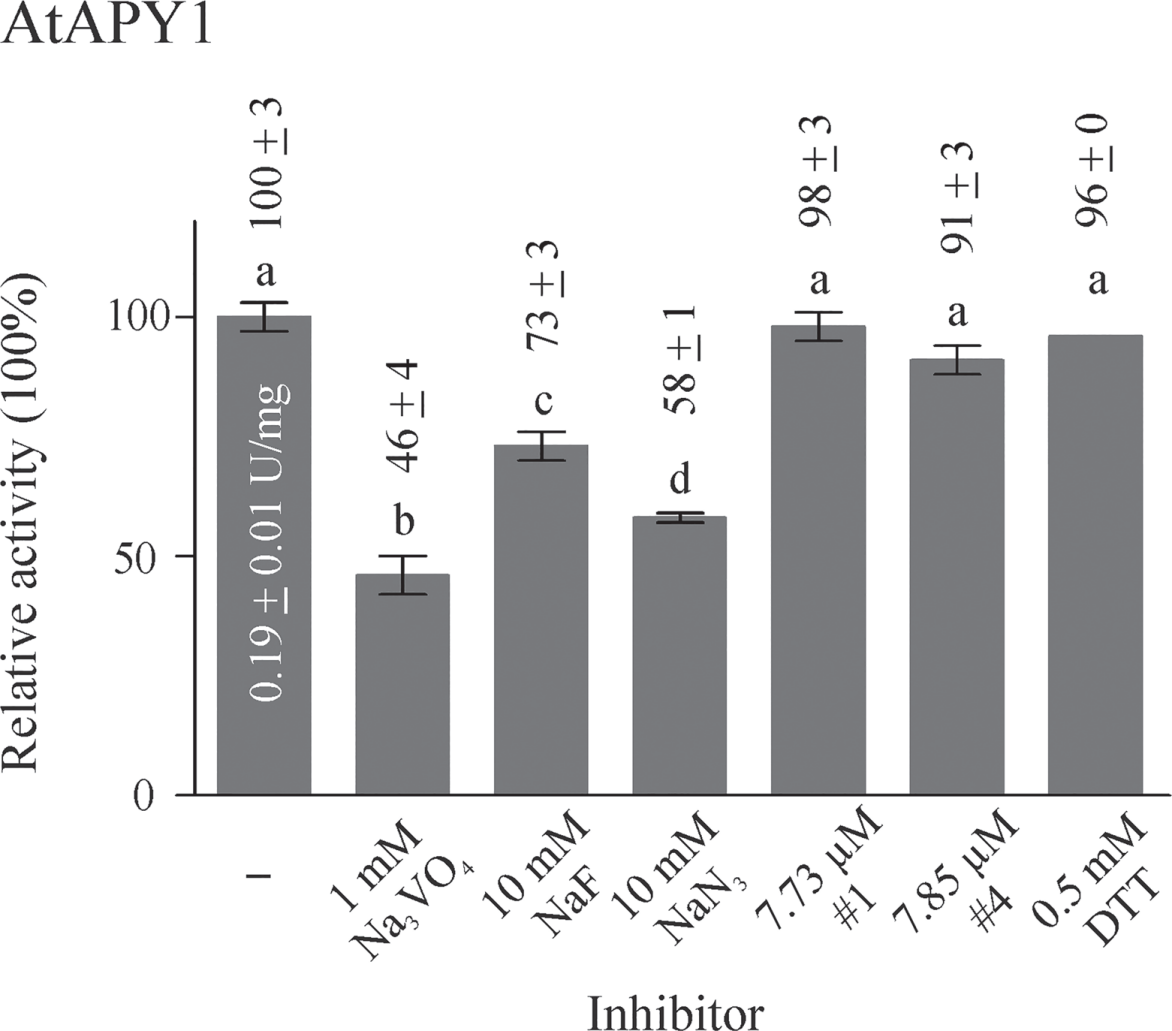
Influence of inhibitors on AtAPY1 activity. Enzyme activities were determined in the presence of 3 mM UDP using the discontinuous apyrase activity assay. The AtAPY1 activity (1 U = 1 μmol P_i_ /min) was measured in the presence of various inhibitors and the activity without inhibitor (-) was defined as 100%. The means + SD of duplicates from one assay are shown. Different letters above the columns indicate mean values that are significantly different from one other (one-way ANOVA and Tukey test; p < 0.05). The data are representative of four assays.

Apyrases are typically inhibited by chelating agents (as confirmed for AtAPY1-GFP in S6 Fig.). Other potential inhibitors are azides and the phosphate analogs orthovanadate (VO_4_^3-^) and fluorides. To test the inhibitory potential of these chemicals, the effect of sodium vanadate, sodium fluoride and sodium azide on AtAPY1 activity was investigated (Fig. 7). Vanadate (1 mM) had the strongest effect, reducing the activity of AtAPY1 by 54%. Fluoride and azide inhibited the activity by 27% and 42%, respectively, at a concentration of 10 mM. The same inhibition pattern was found for AtAPY1-GFP (S7 Fig.).

The three chemicals tested are rather non-specific inhibitors. Vanadate and NaF also inhibit alkaline phosphatases, vanadate also P-type ATPases, and sodium azide inhibits F-type ATPases. With the objective to find apyrase-specific inhibitors, Windsor et al. [60] screened a chemical library of low-molecular-weight compounds, using a commercially available potato apyrase (grade VI; Sigma) as the model NTPDase. Among others, they found the two inhibitors #1 (also published under the name NGXT191) (S1 Fig.) and #4 (S2 Fig.), belonging to the structural class of sulfonamides. Although these inhibitors turned out not to be completely apyrase-specific because they inhibited alkaline phosphatases as well [61], they have become useful tools in deciphering the physiological role of extracellular E-NTPDases in plants [23, 24, 29, 62–64].

First, the inhibitory effect of these two chemicals was tested on a commercially available recombinant potato apyrase. Both compounds reduced the apyrase activity by approximately 40% (S8 Fig.). This result confirmed the previously published inhibitory effects [60], although it is unknown if the potato apyrases analyzed in the previous publication and here are the same, because the commercial suppliers do not provide the sequences of their apyrase products, and many different potato apyrase proteins exist [65].

Next, the inhibitors were tested at the concentration that had previously been shown to reduce the activity of soluble Arabidopsis ecto-apyrase by over 80% compared with the control [23]. The activity of AtAPY1 or AtAPY1-GFP, however, was not affected by the inhibitors at this concentration of 2.5 μg/mL (= 7.73 μM inhibitor #1 and 7.85 μM inhibitor #4, respectively) or by their solvent DMF alone (Fig. 7, S7 Fig.). Obviously, the inhibitors #1 and #4 do not inhibit apyrases in general. This observation was confirmed by others who tested the inhibitor #1 on the recombinant soybean (*Glycine soja*) plasma membrane-bound ecto-apyrase GS52 whose activity remained unaffected by concentrations of up to 30.9 μM (= 10 μg/mL) [66].

Finally, the inhibitory potential of a reducing agent was tested, because the cysteines C87 and C337, C292 and C342 and C397 and C420 (see Fig. 1) were predicted to form disulfide bonds [54]. However, the reductant DTT (0.5 mM) had no effect on either AtAPY1 (Fig. 7) or AtAPY1-GFP (S7 Fig.) activity.

In summary, AtAPY1 and AtAPY1-GFP responded alike to the substances tested. Only the rather non-specific inhibitors vanadate and to a smaller degree fluoride and azide were effective.

### AtAPY1-GFP has highest affinity for GDP

Previously, the nucleoside diphosphates GDP, IDP and UDP were identified as substrates for AtAPY1-GFP [22]. To determine the order of preference, the initial reaction velocities were monitored as a function of substrate concentration in a continuous reaction. This continuous assay involved the coupling of the P_i_-producing AtAPY1-GFP with the P_i_-converting enzyme nucleoside phosphorylase (PNP). The final product AMM absorbs light of 360 nm [38].

In order to ensure that the coupling enzyme PNP never became rate limiting, the initial velocities of AtAPY1-GFP were recorded as a function of AtAPY1-GFP concentration. The reaction rates increased linearly with increasing amounts of AtAPY1-GFP (exemplarily shown for the substrate GDP in S9 Fig.), demonstrating that the reaction conditions were sufficient.

**Fig 8:**
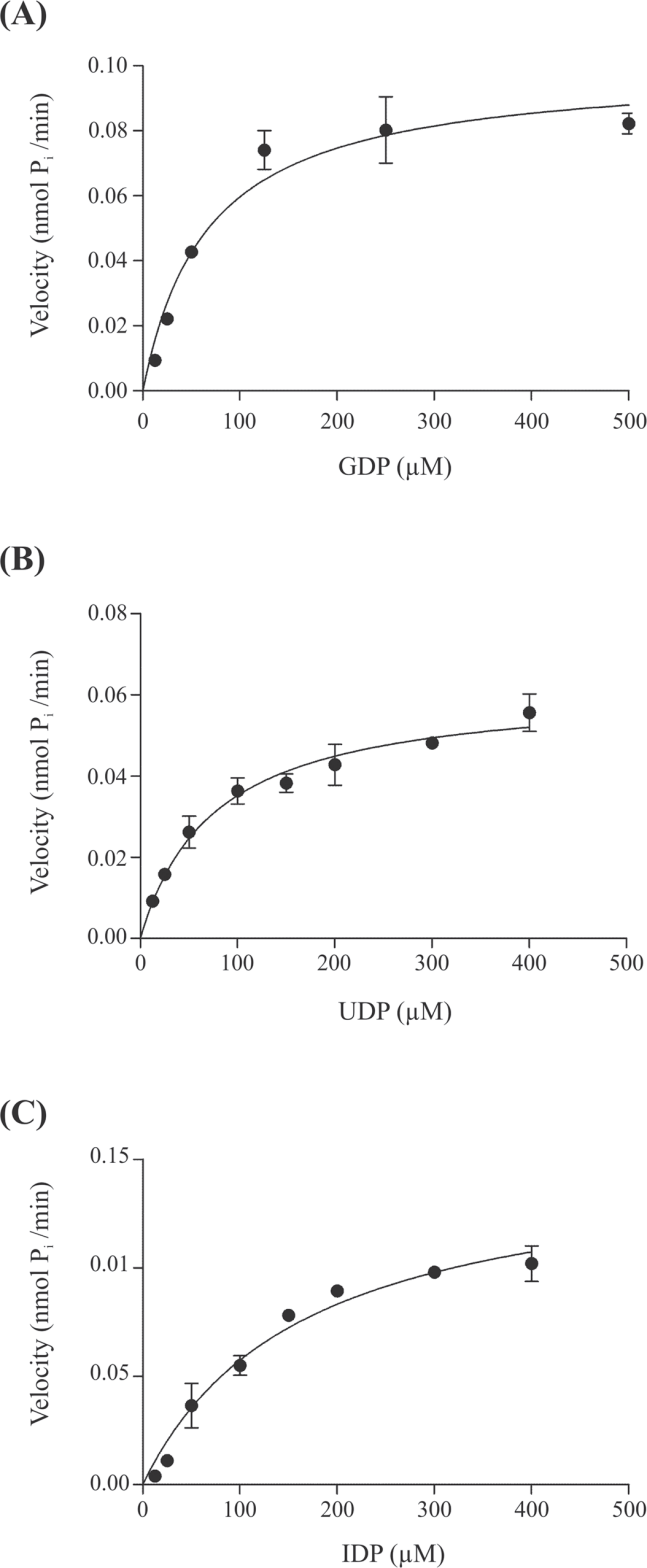
K_m_ values of AtAPY1-GFP for GDP, UDP and IDP. Michaelis-Menten plots of the initial reaction velocities (v) for different concentrations of substrate are shown. Different amounts of AtAPY1-GFP enzyme were used in (A), (B) and (C) as a result of different starting material. The enzyme velocities were determined by the continuous assay. For each substrate concentration, the mean velocity calculated from two parallel reactions was plotted. The initial velocities were linear over time for ≥ 30 min. Each initial velocity was determined from a minimum of 24 data points from this linear phase. The error bars represent the standard deviations of the velocity means. The data set is representative of six (A, B) and three experiments (C), respectively.

Plotting the initial reaction velocities versus the substrate concentrations fit the Michaelis-Menten model for all three substrates tested (Fig. 8A-C). The calculated K_m_ value was 59.7 ± 12.5 μM for GDP, 74.4 ± 9.88 μM for UDP and 166 ± 25.4 μM for IDP (Table 2).

**Table 2:** K_m_ values.

**K_m_ (μM)**	**substrate**
59.7 ± 12.5	GDP^1^
74.4 ± 9.88	UDP^2^
166 ± 25.4	IDP^3^

The mean K_m_ values are listed ± SD. The K_m_ values for GDP, UDP and IDP were all significantly different from each other (p < 0.0001; one-way ANOVA test and Tukey test).

^1^The mean of the K_m_ value was calculated from six separate experiments. The means were not statistically different from each other (p < 0.001; one-way ANOVA). AtAPY1-GFP purified from three different protein extracts (biological repeats) was analyzed. One, two and three separate experiments were run with each protein extract, respectively.

^2^The mean of the K_m_ value was calculated from six separate experiments. The means were not statistically different from each other (p < 0.001; one-way ANOVA). AtAPY1-GFP purified from two different protein extracts (biological repeat) was analyzed. Two and four separate experiments were run with each protein extract, respectively.

^3^The mean of the K_m_ value was calculated from three separate experiments. The means were not statistically different from each other (p < 0.01; one-way ANOVA). AtAPY1-GFP purified from two different protein extracts (biological repeat) was analyzed resulting in one technical and one biological repeat.

The hydrolysis activity was highest for UDP followed by an equally high activity for GDP and IDP [22]. The order of affinities, however, turned out be GDP > UDP >> IDP, rendering AtAPY1-GFP primarily a GDPase.

### Substrate specificity

**Fig 9:**
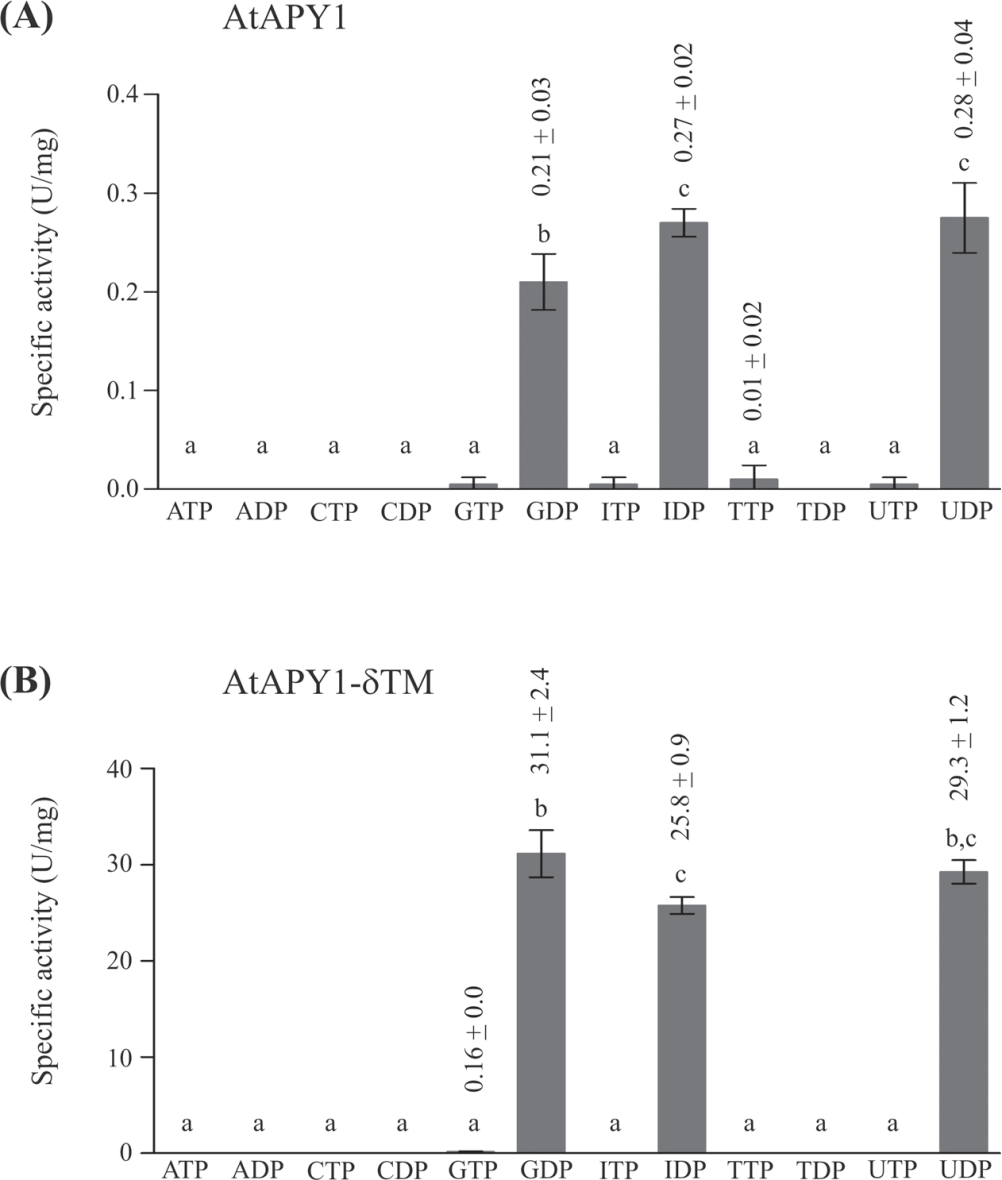
Substrate specificities of AtAPY1 and AtAPY1-δTM. Activities (1 U = 1 μmol P_i_ /min) of AtAPY1 (A) and AtAPY1-δTM (B) were determined in the presence of various substrates (3 mM each) using the discontinuous apyrase activity assay. The means + SD of duplicates from one assay are shown. Different letters above the columns indicate mean values that are significantly different from one other (one-way ANOVA and Tukey test; p < 0.05). Each data set is representative of three independent activity assays.

There is some debate if AtAPY1 has a second localization site in the cell wall where the enzyme would hydrolyze ATP, although it was shown that AtAPY1-GFP does not accept ATP as a substrate [22]. One of the arguments that AtAPY1 might still degrade ATP was that the GFP-tag might interfere with its native substrate specificity. Therefore, a substrate specificity analysis of the GFP-tagless AtAPY1 was conducted. However, as shown in Fig. 9A, only GDP, IDP and UDP were hydrolyzed by AtAPY1, while ATP and other nucleotides were not.

Since AtAPY1 contains a N-terminal region which might serve as a secretory signal peptide as shown for two mammalian homologs called NTPDase6 from man and rat [56, 57], AtAPY1 might exist as a soluble protein as well. For the rat NTPDase6, the substrate specificity was the same for both forms [57], but in the case of the human homolog, only the soluble form was a diphosphatase, while the membrane-bound form hydrolyzed triphosphates as well [56].

To find out if the loss of the TM would also change the substrate specificity of AtAPY1, its soluble form was analyzed. However, the pattern of hydrolysable nucleotides remained the same (Fig. 9B). So the substrate specificity of AtAPY1 with or without TM is limited to the nucleotides GDP, IDP and UDP.

### Activity stimulation by calmodulin

AtAPY1 carries a putative CBS (Fig. 1) and was previously found to bind calmodulin 2 from *A*. *thaliana* (AtCaM2) [20]. However, it had not been investigated yet, if CaM had any effect on AtAPY1 activity.

**Fig 10:**
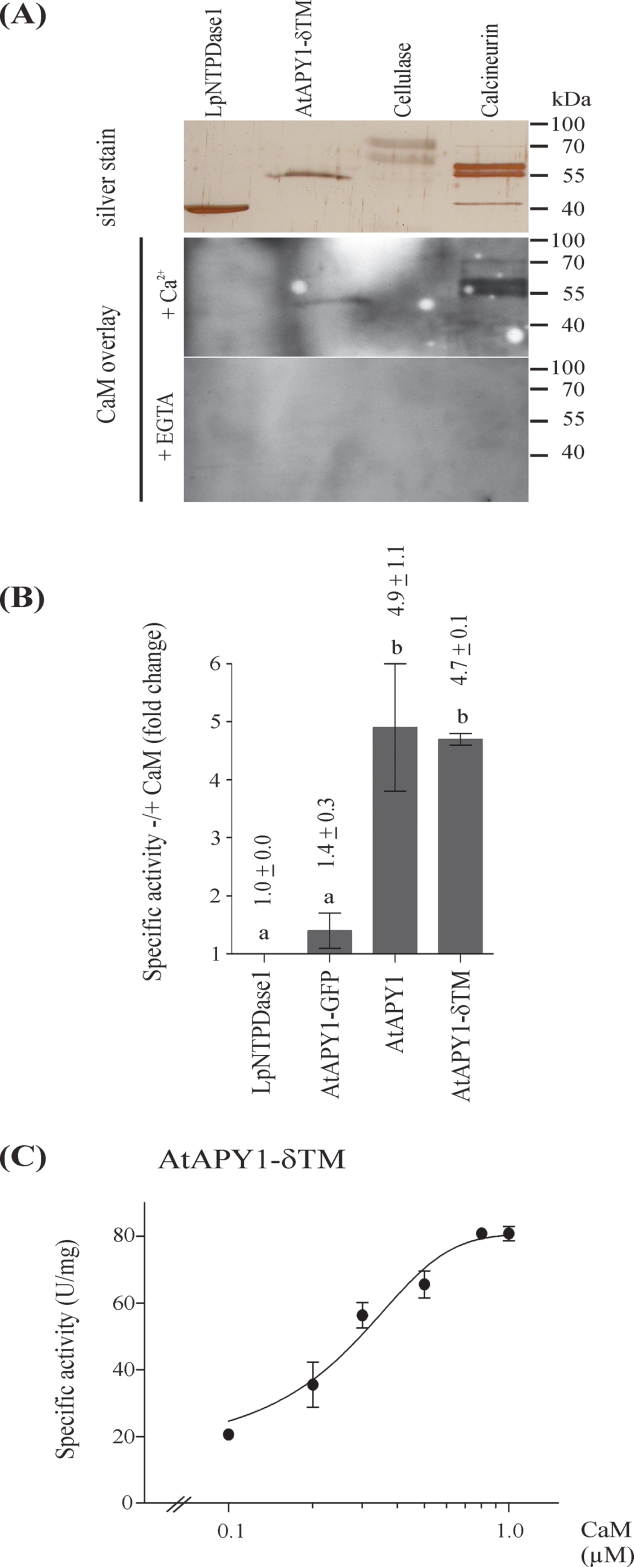
Stimulation of AtAPY1 activity by CaM. (A) Similar amounts (~ 150 ng) of the specified proteins were loaded per lane of a 12% SDS gel, separated by PAGE and subsequently either silver-stained or blotted onto a membrane for a CaM overlay in the presence of either calcium or EGTA. For each of the three applications, all samples were processed in parallel and identical volumes of the same protein sample were loaded. Biotinylated CaM bound by the proteins was detected via chemiluminescence after incubation with alkaline phosphatase coupled to streptavidin. The exposure time for the detection of CaM binding on the Ca^2+^-treated membrane was 1 s versus 60 s for the EGTA-treated membrane based on normalization via a positive control shown in S10 Fig. The results are representative of three independent CaM overlays. (B) The specific activities were determined by the discontinuous assay in the presence of 1 mM CaCl_2_ and 3 mM ADP (LpNTPDase1) or 3 mM UDP (AtAPY1-GFP, AtAPY1, AtAPY1-δTM) and compared with the activities under the same conditions, except for the addition of 0.8 μM CaM (LpNTPDase1) or 1.0 μM CaM (AtAPY1-GFP, AtAPY1, AtAPY1-δTM). Error bars represent the standard deviations of the means in fold activity change by the presence of CaM from two independent activity assays. (C) The specific activity of AtAPY1-δTM was determined by the discontinuous assay in the presence of 3 mM UDP, 1 mM CaCl_2_ and rising concentrations of CaM as indicated. The data were fit to the Hill equation. The means + SD of duplicates from one assay are shown. The result is representative of two independent assays. 1 U = 1 μmol P_i_ /min.

First, the ability of AtAPY1 to bind CaM was confirmed. For this, the same CaM as used previously was recombinantly produced, purified and subjected to biotinylation. The success of the biotinylation reaction was confirmed experimentally (S10 Fig.). AtAPY1-δTM was blotted onto membrane and incubated with the biotinylated AtCaM2 (Fig. 10A). This AtAPY1 version was chosen because relatively pure and large amounts of protein are needed for the CaM-overlay method. Cellulase and *Legionella pneumophila* (Lp) NTPDase1 were included as negative controls, since neither protein contains a putative CBS. The CaM-binding protein-phosphatase calcineurin served as the positive control. As CaM binds its target in a Ca^2+^-dependent manner, the incubation was performed with two membranes in parallel, one in the presence of calcium and the other one without. In addition, the incubation buffer without calcium contained the chelator EGTA. Binding of CaM to a target was visualized with a streptavidin-based assay.

Only AtAPY1-δTM and calcineurin produced signals and only in the presence of calcium, demonstrating that AtAPY1-δTM bound CaM specifically. To ensure that the exposure time of the EGTA-treated membrane was long enough, both membranes carried the same amount of a biotinylated control protein (= Snap-Biotin). The exposure times of the calcium- and EGTA-treated membrane were chosen so that the intensity of the Snap-Biotin signal from each was comparable (S10 Fig.).

Next, activity assays were performed to see if CaM binding would evoke stimulation or repression of enzyme activity. All three AtAPY1 versions, AtAPY1-GFP, AtAPY1 and AtAPY1-δTM, were included. LpNTPDase1 served as the negative control which was not stimulated by the addition of calmodulin (Fig. 10B, S11 Fig.). The activity of AtAPY1-GFP increased in the presence of calmodulin, but the increase was not statistically significant (Fig. 10B, S11 Fig.). This result was unexpected, because AtAPY1 can bind CaM in vitro. Obviously, AtAPY1-GFP cannot, whether due to interference from the GFP-tag or from the tethering to the plate well bottom or due to some other reason was not further investigated in this study. AtAPY1 and AtAPY1-δTM, however, were stimulated by calmodulin, both about 5-fold (Fig. 10B), compared with the activity in the presence of calcium alone (S11 Fig.). The stimulation by CaM was in the same range as documented previously for a pea NTPDase [33] and confirmed here (S12 Fig.). These findings show that both the membranous and soluble AtAPY1 are amenable to regulation by CaM.

The CaM-stimulation kinetics in the presence of calcium were analyzed in more detail using the purified AtAPY1-δTM protein and various concentrations of CaM from 0.1–1 μM (Fig. 10C). The CaM-activation curve shows that small changes in the concentration of CaM led to a big increase in enzyme activity. This observed positive co-operativity opens the possibility of an efficient regulation of AtAPY1-δTM by CaM in vivo.

**Fig 11:**
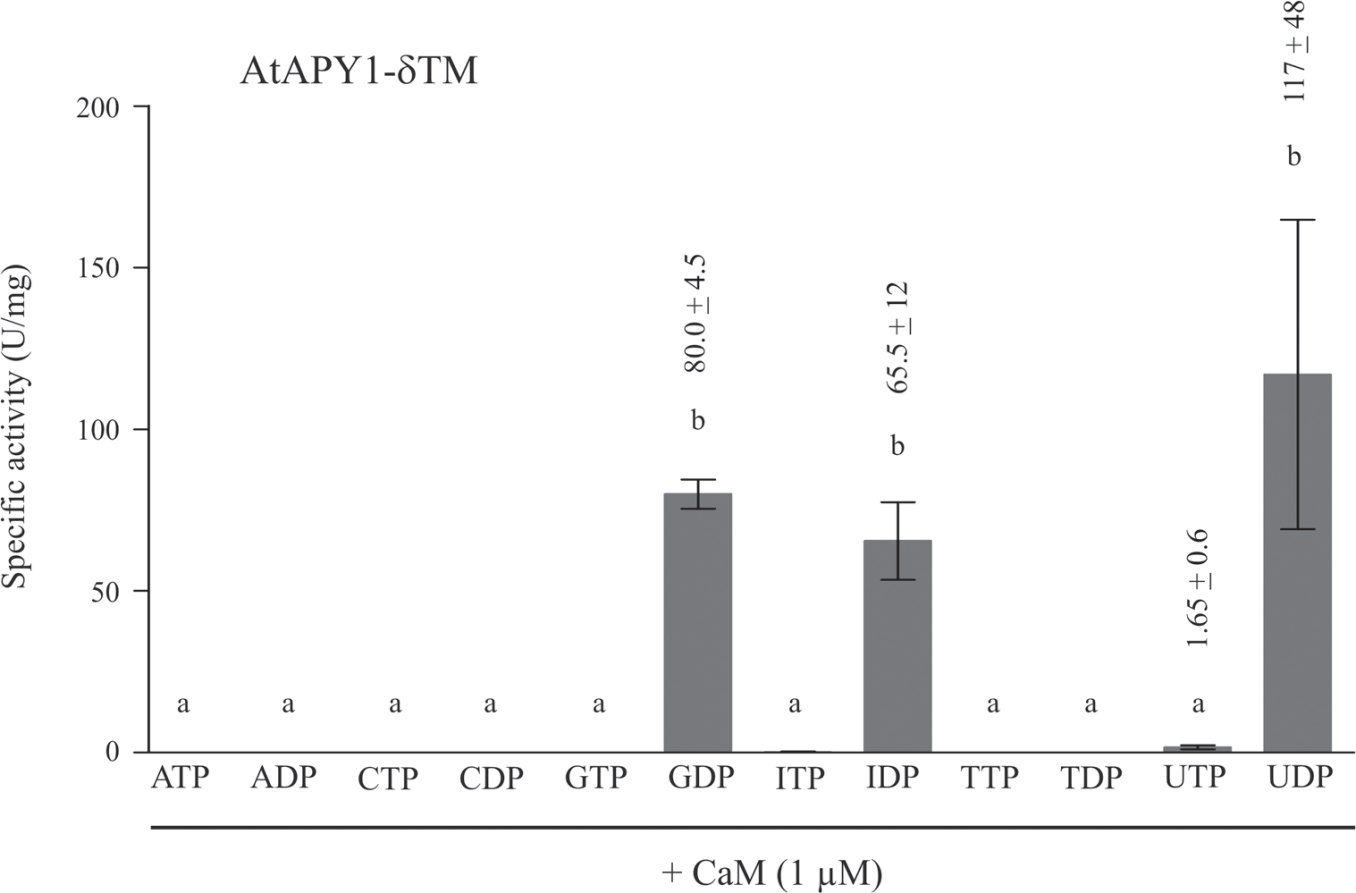
Substrate specificity of AtAPY1-δTM in the presence of CaM. Activities (1 U = 1 μmol P_i_ /min) of AtAPY1-δTM were determined in the presence of various substrates (3 mM each) and 1 μM CaM using the discontinuous apyrase activity assay. The means + SD of three independent assays are shown. Different letters above the columns indicate mean values that are significantly different from one other (one-way ANOVA and Tukey test; p < 0.05).

Whether the CaM regulation is physiologically relevant, however, cannot be answered by the biochemical data. A possible scenario of biological importance other than the impact on enzyme activity would be a change in substrate specificity. CaM is primarily regarded a cytosolic protein, but it also occurs in other compartments, e. g. the extracellular space [67]. Therefore, if AtAPY1 were indeed secreted in planta, CaM would be available to modify its substrate specificity, for example to hydrolyze ATP or ADP. To test this hypothetical scenario, the substrate specificity of AtAPY1-δTM was assayed in the presence of calcium and calmodulin (Fig. 11). Twelve different nucleotides were investigated, but GDP, IDP and UDP remained the only hydrolysable substrates.

### Structural model of the ecto-domain

**Fig 12:**
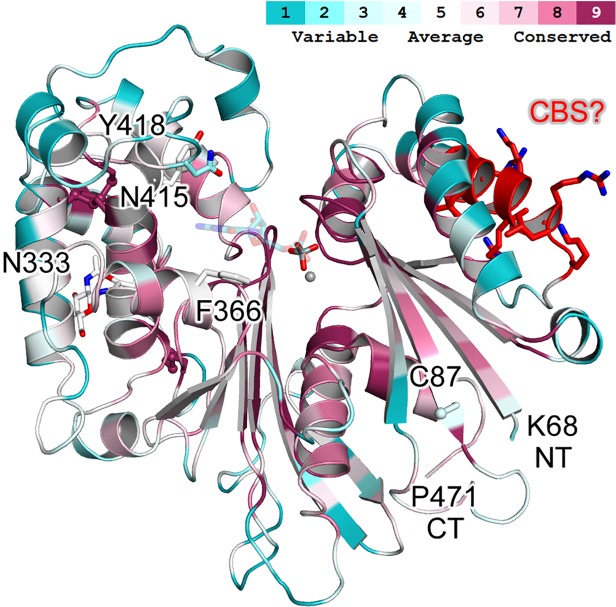
Putative three-dimensional structural model of the AtAPY1 ecto-domain. The structural model was colored using the ConSurf server [83] and 300 random NTPDase sequences sharing between 20 and 80% sequence identity. The N-terminal (NT) and C-terminal (CT) residue are marked. Three conserved cysteine bridges are predicted for the C-terminal lobe, while the non-conserved C87 is likely to be free. A single N-acetyl glucosamine group is drawn to indicate the solvent-exposed position of the single potential N-glycosylation site N333 in the back. Next to the active site Mg^2+^ ion (gray sphere) a vanadate ion is shown in the active site to highlight its likely competitive inhibition mechanism. The putative CaM-binding site (CBS) ^169^VRELLKGRSRLK^180^ is depicted as red sticks. An approximate binding mode for GDP is shown in half transparency. F366 and N415 are strong candidates for base sandwich binding. Y418 might be involved in base binding as well but would have to adopt a different side chain conformation than that assigned by the MODELLER program [48].

A three-dimensional putative structural model of the AtAPY1 catalytic domain was generated, using the ecto-domain structure of rat NTPDase2 (RnNTPDase2) as a template [40]. The resulting model contains residues K68 to P471 of AtAPY1 (Fig. 1) which covers the sequence of AtAPY1-δTM except for the first and terminal proline (P67, P471). In spite of only 23% sequence identity, a strong conservation of the catalytic residues that line the active site cleft between the two lobes of the protein is evident (Fig. 12). The model shows that the putative CBS is easily accessible for Ca^2+^-activated CaM. Similarly, the sole potential N-glycosylation site at N333 is well solvent-exposed and therefore likely to become glycosylated. The model further confirms that three cysteine bridges are formed as predicted by the DiANNA web server [54], however, the model’s prediction of the bonds is different: C292-C322, C337-C342, and C397-C420. Furthermore, the cysteine residue at position 87 is likely to exist as a free sulfhydryl according to the model.

Fig. 12 gives an explanation for the strong inhibitory effect of vanadate found experimentally (Fig. 7, S7 Fig.). Vanadate and other phosphate mimics such as sulfate, ortho-molybdate and ortho-tungstate can bind to the substrate's terminal phosphate binding site in the active site cleft between the two lobes of the protein [34, 45]. In addition, the flexible bond lengths and geometry of vanadate allow it to adopt geometry reminiscent of the transition state, hence further increasing its competitive inhibitory effect.

Our kinetic analyses showed that AtAPY1-GFP had the highest affinity towards GDP (Fig. 8, Table 2). Therefore, an approximate binding mode for the substrate GDP was modeled (Fig. 12). The position and geometry of GDP is based on complex structures of RnNTPDase2 with the ADP analog and the GTP analog GMPPNP [34, 41, 45]. Due to the low sequence conservation in the nucleoside binding region, the predictive power is limited and no substrate docking was performed. Nevertheless, F366 and N415 are strong candidates for base sandwich binding. Y418 most certainly corresponds to Y398 of RnNTPDase2 and Y350 of LpNTPDase1 which are involved in in-plane base binding [40, 41]. However, whereas in RnNTPDase2 and LpNTPDase1 the tyrosine is directly adjacent to the second one of the two canonical disulfide bridges, it is shifted one residue position in AtAPY1 (a leucine is inserted). It is tempting to speculate that this shift is linked to the high nucleoside diphosphate specificity and/or base specificity.

## Discussion

### Plant Golgi E-NTPDases

Among the family of E-NTPDases, the focus of biochemical characterization has been on extracellular E-NTPDases, mainly from mammalian species. Fewer Golgi apyrases have been characterized biochemically so far, and only two of them were from plants: A 50-kDa apyrase from sycamore (*Acer pseudoplatanus*) [68] and an IDPase from rice (*Oryza sativa*) [69]. The corresponding protein or gene sequences of these two Golgi apyrases, however, were not identified, but their substrate specificity and the stimulation of their activity by a wide range of divalent ions make them likely members of the E-NTPDase family. Nevertheless, AtAPY1 is the first plant Golgi apyrase, for which enzymatic properties were unequivocally linked to a protein with known sequence.

### Comparison of the enzymatic properties of AtAPY1, AtAPY1-δTM and AtAPY1-GFP

The enzymatic properties of the three AtAPY1 variants were very similar, if not identical. This is not self-evident considering that a larger region was either removed, in the case of AtAPY1-δTM, or added, in the form of a GFP-tag. Except for the response to Zn^2+^ and CaM, the biochemical analysis of AtAPY1-GFP indicated that the tag was not changing the properties of AtAPY1 in agreement with previous genetic complementation experiments [22]. The finding that AtAPY1-GFP represents most of the enzymatic properties of the native enzyme could make the GFP-multiTrap system a useful tool to efficiently screen for inhibitors and activators.

### Substrate specificity of Golgi E-NTPDases

**Fig 13:**
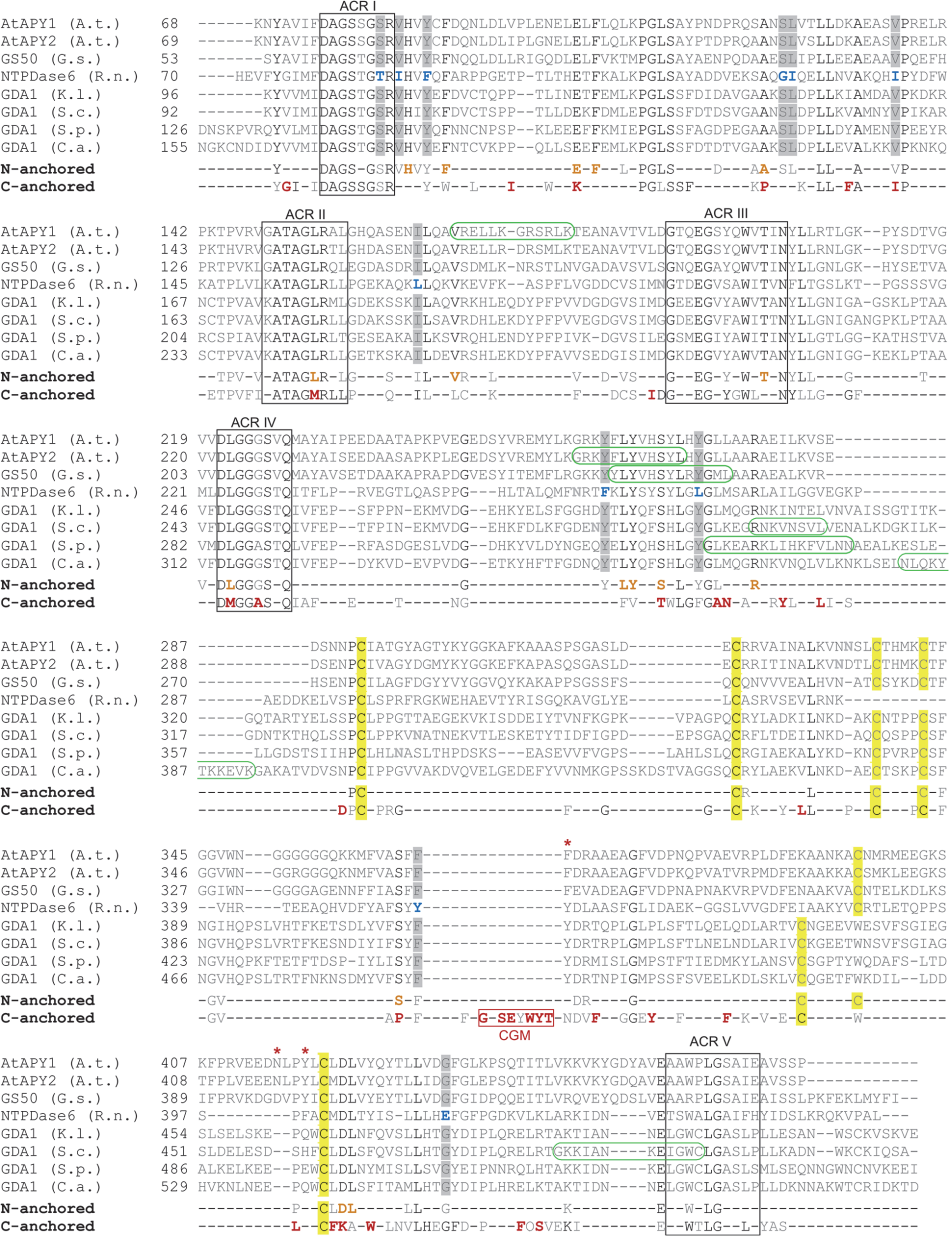
Alignment of single-pass type II membrane Golgi E-NTPDases. The sequence alignment was generated using all known Golgi E-NTPDase sequences which contain one TM near the N-terminus (= N-anchored): AtAPY1 from *A*. *thaliana* (A. t.) [UniProtKB: Q9SQG2], AtAPY2 from A. t. [UniProtKB: Q9SPM5], GS50 from *Glycine soja* (G. s) [UniProtKB: Q9FVC3], NTPDase6 from *Rattus norvegicus* (R. n.) [UniProtKB: Q9ER3], GDA1 from *Kluyveromyces lactis* (K. l.) [UniProtKB: Q9HEM6], GDA1 from *Saccharomyces cerevisiae* (S. c.) [UniProtKB: P32621], GDA1 from *Schizosaccharomyces pombe* (S. p.) [UniProtKB: Q9UT35] and GDA1 from *Candida albicans* (C. a.) [UniProtKB: Q8TGH6]. The sequences (without the N-terminal regions) are presented in descending order of identity to AtAPY1: AtAPY1 and AtAPY2 share the highest (87% identity) and AtAPY1 and C. a. GDA1 the lowest identity (24%). Their consensus is presented as “N-anchored”. The ACRs are boxed. Highly conserved cysteines are highlighted in yellow. The candidate amino acids for substrate binding in AtAPY1 (see Fig. 12) are labeled with red asterisks. Differences between the NTPDase6 and the other seven N-anchored Golgi E-NTPDases are shown in blue and highlighted in gray, respectively. A putative CBS is circled in green. The “C-anchored” consensus from all known Golgi E-NTPDase sequences which contain one TM near the C-terminus (= C-anchored) was generated separately: NTP-1 from *C*. *elegans* (C. e.) [UniProtKB: Q18411], S. p. YND1 [UniProtKB: Q9USP2], S. c. YND1 [UniProtKB: P40009] and K. l. YND1 [UniProtKB: Q70KY5]. For the extraction of the consensus sequences, the sequences of the regions from ACR I to ACR V were evaluated, omitting the respective N- and C-terminal parts with the TMs from the analysis. Amino acids present in all members of the N- and/or C-anchored class are in black. If the amino acids are exclusively present in all members of one class, they appear in orange or red, respectively. The 8-aa region marked CGM (C-anchored Golgi NTPDase conserved motif) is unique to C-anchored Golgi E-NTPDases.

Regarding substrate specificity, there are clear differences between Golgi E-NTPDases with a TM near the N-terminus (= N-anchored) or the C-terminus (= C-anchored). AtAPY1 belongs to the N-anchored Golgi apyrases, and the complete alignment of all eight confirmed members across species is shown in Fig. 13. While all C-anchored E-NTPDases hydrolyze ADP or ATP [12, 14, 15, 70, 71], all N-anchored NTPDases do not [14, 22, 72, 73]. The substrate specificity of AtAPY1 presented here fits this classification.

To identify obvious sequence differences which may explain the different substrate spectra between these two classes, their consensus sequences were compared (Fig. 13). The amino acids proposed responsible for GDP binding in AtAPY1 are residues F366, N415 and Y418 (Fig. 12). Strikingly, strong differences between the two consensus sequences occur near these amino acids. C-anchored E-NTPDases exhibit an insertion of several highly conserved amino acids (= C-anchored Golgi E-NTPDase conserved motif) before F366 of AtAPY1. In addition, the C-anchored E-NTPDases also differ greatly with the N-anchored consensus sequence shortly after the Y418 of AtAPY1. These sequence differences may serve as starting points to study the differences in substrate specificity.

### Stimulation of AtAPY1 activity by CaM

The finding that AtAPY1 activity was enhanced by CaM suggests that this is a regulatory mode of this enzyme with physiological implications. However, so far no other Golgi-resident CaM-binding proteins have been identified. In animals, the δ2 isoform of the CaM-dependent kinase II is often ambiguously referred to as a Golgi enzyme, but it was localized at the Golgi and not inside [74]. Regarding CaM, none of the various isoforms have been localized in the Golgi either. However, not all CaM isoforms and CaM-binding proteins have been localized so far.

There is one known Arabidopsis CaM-binding protein in the ER, a subunit of the ER-resident glucosidase II, which was identified as a CaM-binding protein [75]. From this it can be inferred that CaM exists in the ER and from this, in turn, it can be imagined that CaM exists in its closely associated compartment, the Golgi, as well.

So far, only one other plant NTPDase has been demonstrated to be stimulated by CaM [33] raising the possibility that this regulatory feature might be plant-specific. However, it could also be that the possible regulation of NTPDases by CaM is scarcely documented, because it has been simply overlooked as an option. The bioinformatics analysis at least predicts CBS in other Golgi E-NTPDases from other species (Fig. 13) warranting experimental exploration.

### AtAPY1 and purinergic signaling

The discovery that eATP elicits a spike in cytosolic Ca^2+^ concentration in plants [76, 77], sparked research efforts to unravel the underlying signaling pathway. AtAPY1 and AtAPY2 emerged as the prime candidates for the NTPDase activity breaking down eATP [27]. Genetic evidence was provided by transgenic Arabidopsis lines overexpressing *AtAPY2*. Application of ATP on leaves of WT and the overexpressing plants effected the accumulation of superoxide in both genotypes with the difference, however, that the accumulation was much less in the overexpressors [78]. This result implied that the overexpression of *AtAPY2* lowered the concentration of eATP. To test this, a poorly hydrolysable ATP analog was applied, and indeed the difference in superoxide production between the two genotypes disappeared [78]. These experiments dealt with AtAPY2, but since previous genetic complementation had shown that *AtAPY1* was functionally redundant with *AtAPY2* [25, 26], AtAPY1 was linked to purinergic signaling, too. However, the substrate specificity presented here conflicts with AtAPY1 as a regulator of ATP signals.

One reconciliatory explanation would be that AtAPY1 requires certain post-translational modifications or binding partners that activate its ATP-degrading mode. However, the AtAPY1 proteins analyzed here were synthesized in a host that provides eukaryotic post-translational modifications. In the case of AtAPY1-GFP, the enzyme was even extracted from its native source, warranting possibly important post-translational changes and even supplying possible binding partners. Nevertheless, a small proportion of AtAPY1-GFP bound to a substrate specificity modifier could remain undetected in the substrate specificity analyses.

However, even if this scenario were true, it would not explain the nature of the extracellular ATP-degrading NTPDase activity that was previously observed to be inhibited by the chemicals NGXT191 and/or 194 [23, 62]. Therefore, a more likely situation which is discussed in more detail elsewhere [79] seems to be that the observed eATP hydrolysis stems from a different E-NTPDase. There are five more Arabidopsis genes encoding E-NTPDases, two of which are predicted to have two TMs [80] which is a typical feature of ATP-degrading E-NTPDases in mammals [1]. Furthermore, the list of candidates grows even longer if various isoforms of AtAPY2 and AtAPY3 from possible alternative splicing events are considered [80].

Along those lines, AtAPY2 instead of AtAPY1 could be the sought-after enzyme responsible for eATP hydrolysis, because even though the genetic complementation experiments [25, 26] demonstrated that AtAPY1 and AtAPY2 can take over each other’s function, AtAPY2 might hydrolyze ATP (and possibly other nucleotides, too) in addition to the substrates hydrolyzed by AtAPY1.

However, the putative structural model of the AtAPY1 ecto-domain implicates the amino acids F366, N415 and Y418 of the catalytic center in substrate binding (Fig. 12), and these exact same residues are present in AtAPY2 (Fig. 13). This strong conservation argues against a different substrate specificity of AtAPY2.

A study measuring ATP levels in the liquid culture medium of WT and RNA interference (RNAi) mutant seedlings also questions the idea that AtAPY2 instead of AtAPY1 is responsible for eATP breakdown [81]. These mutants lacked a functional *AtAPY2* gene, but carried an intact *AtAPY1* gene. The amounts of *AtAPY1* transcripts expressed by this intact gene could be drastically lowered on demand by inducing RNAi [23]. eATP levels were the same in the medium of WT and non-induced RNAi mutants. Only when RNAi was induced, did the eATP concentrations rise significantly in the medium with the mutants compared with the WT controls. This result clearly pointed to AtAPY1 as the hydrolyser of eATP.

The conflicting data of AtAPY1’s substrate specificity and the correlation of eATP levels going up with suppression of *AtAPY1* transcript abundance are puzzling, but can be reconciled if AtAPY1 regulates the concentration of eATP indirectly. This could be achieved through a role in glycosylation (as will be outlined in below), leading to pleiotropic effects. As an example, defective glycosylation was shown to increase salt sensitivity in plants, because proteins conferring salt resistance were compromised in their activity due to improper maturation in the Golgi [82]. Similarly, AtAPY1 could be crucial for the glycosylation state of a protein which is involved in ATP delivery or eATP hydrolysis and which depends on glycosylation to perform its function.

### AtAPY1 and glycosylation

The in-vivo functions of the Golgi apyrases guanosine diphosphatase 1 protein (Gda1p) and yeast nucleoside diphosphatase 1 (Ynd1p) from the yeast *Saccharomyces cerevisiae* (S. c.) have been studied in greater detail. The null mutant *Δgda1* [ 13 ] and *Δynd1* [ 15 ] both displayed N- and O-linked glycosylation defects. The double mutant *Δgda1Δynd1* led to cell aggregation and slow growth, presumably due to compromised cell wall integrity [ 15 ]. These mutant phenotypes have become a popular tool to test the function of other Golgi apyrases. The GDA1 homologs from *Kluyveromyces lactis* (Kl) [ 17 ] and *Candida albicans* [ 16 ] each suppressed the N- and O-linked glycosylation defects in Sc*Δgda1*. The apyrase MIG-23 from *C*. *elegans* was also shown to rescue the growth defects of Sc*Δgda1Δynd1* mutants [ 18 ], and therefore linked to a role in glycosylation.

The same approach was chosen for *AtAPY1* which complemented the Sc*Δgda1* mutant and recovered N-glycosylation of a reporter protein [ 31 ]. This complementation result is a strong indication that AtAPY1 also supports glycosylation in the Golgi of Arabidopsis, however, rescue of the Sc*Δgda1* mutant phenotype does not necessarily imply the same function in a different organism. For example, the loss of *KlGDA1* did not cause N-glycosylation defects in *K*. *lactis*, but a knock-in of this gene still rescued these defects in Sc*Δgda1* [ 17 ].

Another piece of supporting evidence for AtAPY1’s possible role in glycosylation comes from a microarray data analysis, which compared the transcript abundance differences between WT and the aforementioned RNAi mutants. In this study, genes involved with UDP-glycosyltransferase activity were significantly downregulated in the induced RNAi mutants [ 81 ]. In the Golgi, UDP-glycosyltransferases are the enzymes that transfer the sugar from a UDP-sugar to an acceptor molecule. All of this circumstantial evidence points to a role of AtAPY1 in glycosylation, but direct confirmation is needed.

## Conclusions

The enzymatic properties of AtAPY1, mainly the absence of catalytic hydrolysis of ATP and ADP, counter a direct role of AtAPY1 as an extracellular E-NTPDase regulating purinergic signals, but rather indicate that it is a Golgi-resident nucleoside diphosphatase (EC 3.6.1.6), possibly involved in glycosylation processes.

## Supporting Information

S1 Fig Chemical structure of inhibitor #1 (NGXT191). (TIF)

S2 Fig Chemical structure of inhibitor #4 (NGXT194). (TIF)

S3 Fig Amino acid sequences encoded by the constructs for AtAPY1-GFP, AtAPY1 and AtAPY1-δTM. Letters in bold designate amino acids appearing in the native AtAPY1 protein sequence, while letters in italics denote introduced amino acids. Orange coloring highlights the sequence of the secretory signal peptide which is cleaved off during secretion.
(TIF)

S4 Fig Reproducibility of AtAPY1-GFP extraction with the GFP-multiTrap plate methodology. Forty-four μg of a crude protein extract from WT (white bars) or *AtAPY1-GFP* expressing (gray bars) seedlings were added per well of one GFP-multiTrap plate for immobilization of AtAPY1-GFP. The amount of immobilized protein was determined as described in Materials and Methods. Data from eight wells are shown. The error bars represent the standard deviations of the mean calculated from four fluorescence measurements per well. The limit of quantitation (solid line) and the detection limit (dashed line) are defined as ten and three times the SD of the background (= average fluorescence of > 10 Tris-MES buffer measurements), respectively. Identical letters mark no statistically significant difference in the amounts of bound GFP per well (one-way ANOVA and Tukey test; p < 0.001). The difference in bound GFP between the AtAPY1-GFP containing samples and the WT controls was statistically significant (p < 0.0001). The data are representative of all GFP immobilization experiments.
(TIF)

S5 Fig Western blot analysis of purification fractions of AtAPY1-δTM. Several of the AtAPY1-δTM purification fractions shown in Fig. 4B (right panel) were subjected to Western blot analyses. Half of each fraction volume used for Fig. 4B (right panel) was loaded on a 5–12% gradient gel, so the protein amount separated in lane E1 equals about 35 ng. S, supernatant; FT, flow through; E1, eluate 1.
(TIF)

S6 Fig Dependence of AtAPY1-GFP activity on divalent ions as cofactors. Discontinuous activity assays of AtAPY1-GFP in the presence of the substrate UDP (3 mM) were performed. Error bars represent standard deviations of the means of duplicates from one assay. The data are representative of three activity assays with independent protein extracts. (A) The activity was measured in the presence of either 1 mM CaCl_2_, CuCl_2_, MgCl_2_, MnCl_2_, NiCl_2_ or ZnCl_2_. The control (-) shows the activity without the addition of any divalent ions. Different letters above the columns indicate mean values that are significantly different from one other (one-way ANOVA and Tukey test; p < 0.01). (B) The activity was determined without the addition of any divalent ions. The assay buffer contained no (-) or 1 mM EDTA. The asterisk (*) indicates that the means are statistically different (p < 0.05; two-tailed unpaired t-test).
(TIF)

S7 Fig Effect of ATPase and apyrase inhibitors on AtAPY1-GFP activity. The AtAPY1-GFP activity was measured in the presence of various inhibitors and UDP (3 mM) as substrate by using the discontinuous assay. (A) The activity without inhibitor (-) of 0.121 + 0.005 nmol P_i_ /min was defined as 100%. Different letters above the columns indicate mean values that are significantly different from one other (one-way ANOVA and Tukey test; p < 0.05). The means + SD of duplicates from one assay are shown. The data are representative of three separate experiments with three different protein extracts. (B) The activity without inhibitor (-) of 0.053 + 0.014 nmol P_i_ /min was defined as 100%. The concentrations of 7.73 μM inhibitor #1 and 7.85 μM inhibitor #4 equal 2.5 μg/mL each. The means + SD of duplicates from one assay are shown. As a control, the activity was determined in the presence of the inhibitor solvent (1% DMF). Data are representative of four independent experiments. Mean values are not significantly different from one other (one-way ANOVA and Tukey test; p > 0.05).
(TIF)

S8 Fig Inhibition of potato apyrase by inhibitors #1 and #4. The effects of the inhibitors #1 and #4 on the activity of potato (*Solanum tuberosum*) apyrase (= StNTPDase) were tested in the presence of ATP (3 mM) as substrate by the discontinuous assay. The concentrations of 7.73 μM inhibitor #1 and 7.85 μM inhibitor #4 equal 2.5 μg/mL each. The activity without inhibitor (-) was defined as 100%. 1 U = 1 μmol P_i_ /min. The means + SD of duplicates from one assay are shown. Both mean values of the inhibitor-treated samples are significantly different from the control (indicated by different letters; one-way ANOVA and Tukey test; p < 0.001), but not from each other (one-way ANOVA and Tukey test; p > 0.05). Data are representative of four independent experiments.
(TIF)

S9 Fig Initial velocities of AtAPY1-GFP as a function of its concentration. The activity of AtAPY1-GFP was measured with the continuous assay in the presence of 500 μM GDP. The means + SD of two independent reactions are plotted. Individual GFP-multiTrap wells were incubated with various volumes of the same protein extract. One arbitrary unit (AU) is defined as the amount of AtAPY1-GFP immobilized from 100 μL of protein extract.
(TIF)

S10 Fig CaM binding of AtAPY1-δTM in CaM overlay. The gel and exposed membranes of the CaM overlays shown in Fig. 10A are presented in full, including all controls. Snap-Biotin (50 ng loaded per lane; framed by dashed lines) represents O^6^-alkylguanine-DNA alkyltransferase which had been covalently auto-biotinylated [84] as described in Materials and Methods and was used as a positive control for the streptavidin-based detection. The exposure time of the EGTA-treated control membrane was chosen so that the signal intensity of the Snap-Biotin band (marked by a yellow arrowhead) was at least as strong as in the exposure of the Ca^2+^-treated membrane. For the CaM overlays, AtCaM2 was biotinylated (= CaM-Biotin) as outlined in Materials and Methods. CaM-Biotin (150 ng each) was analyzed by silver staining and CaM overlay to check its purity and successful biotinylation, respectively. The streptavidin-based detection of CaM-Biotin is indicated by a black arrowhead.
(TIF)

S11 Fig Effect of CaM on activity of LpNTPDase1, AtAPY1-GFP, AtAPY1 and AtAPY1-δTM. Discontinuous apyrase activity assays were performed in the presence of the substrate 3 mM ADP (A) or 3 mM UDP (B, C, D). The concentrations of Ca^2+^ and CaM are indicated. 1 U = 1 μmol P_i_ /min. The means + SD of duplicates from one assay are shown. The data are representative of two (A, D), three (B), four (C) activity assays. Different letters above the columns indicate mean values that are significantly different from one other (one-way ANOVA and Tukey test; p < 0.05).
(TIF)

S12 Fig Stimulation of pea apyrase activity by CaM. The activity of pea apyrase [33] was measured in the presence of 1 mM CaCl_2_, the substrate ATP (3 mM) and CaM by the discontinuous assay. The first column (-) shows the activity in the absence of CaM. The means + SD of two reactions run in parallel are shown. The asterisk indicates that the activity with CaM is significantly higher than without (Student’s t-test; p < 0.05). The data are representative of three activity assays.
(TIF)
