# Supplementary figures and images for: The Biochemical Properties of the Arabidopsis Ecto-Nucleoside Triphosphate Diphosphohydrolase AtAPY1 Contradict a Direct Role in Purinergic Signaling
Source: PLoS One. 2015 Mar 30;10(3):e0115832. doi: 10.1371/journal.pone.0115832 (PMC4379058; doi:10.1371/journal.pone.0115832)

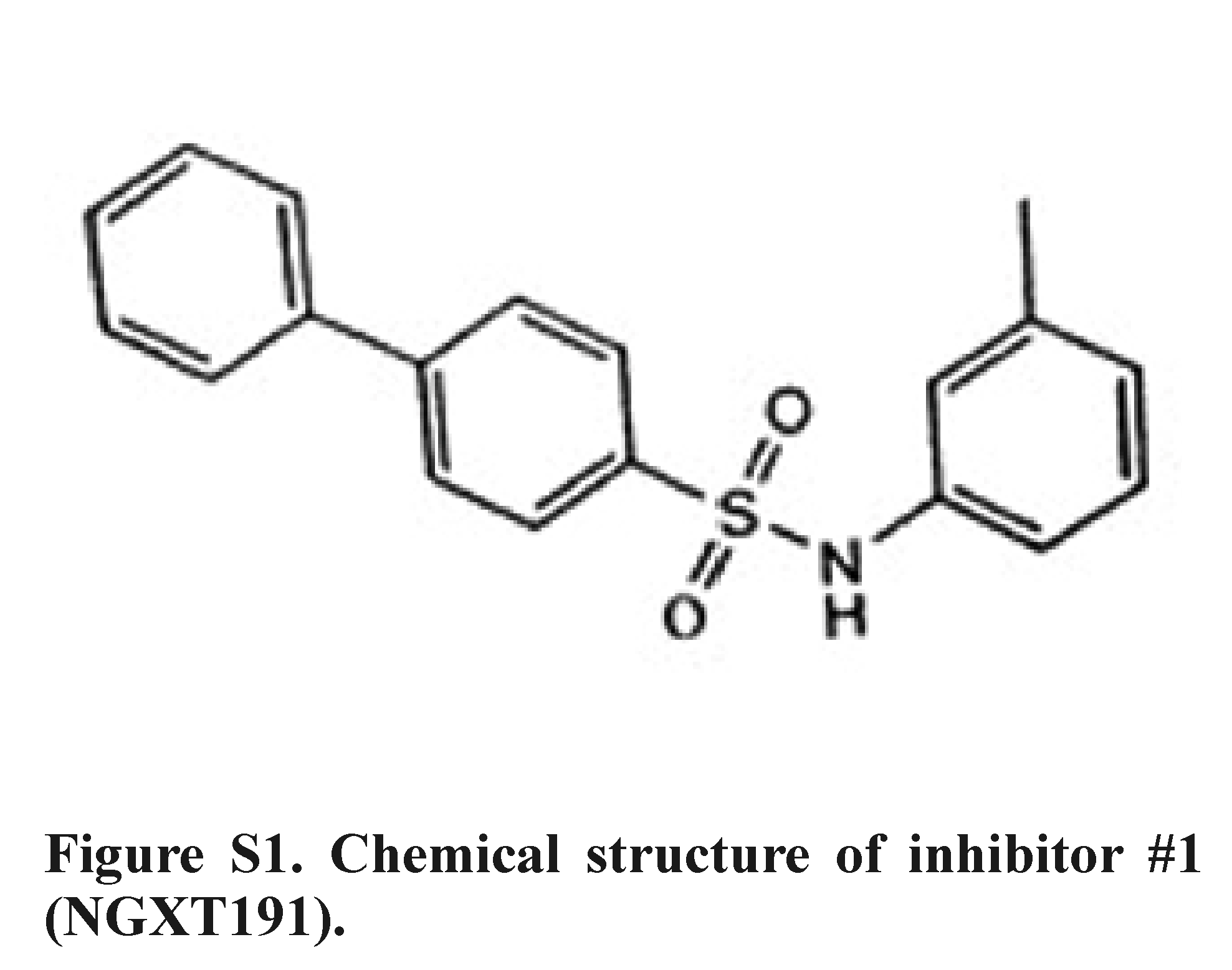

Supplement: S1 Fig — (TIF) [file pone.0115832.s001.tif]

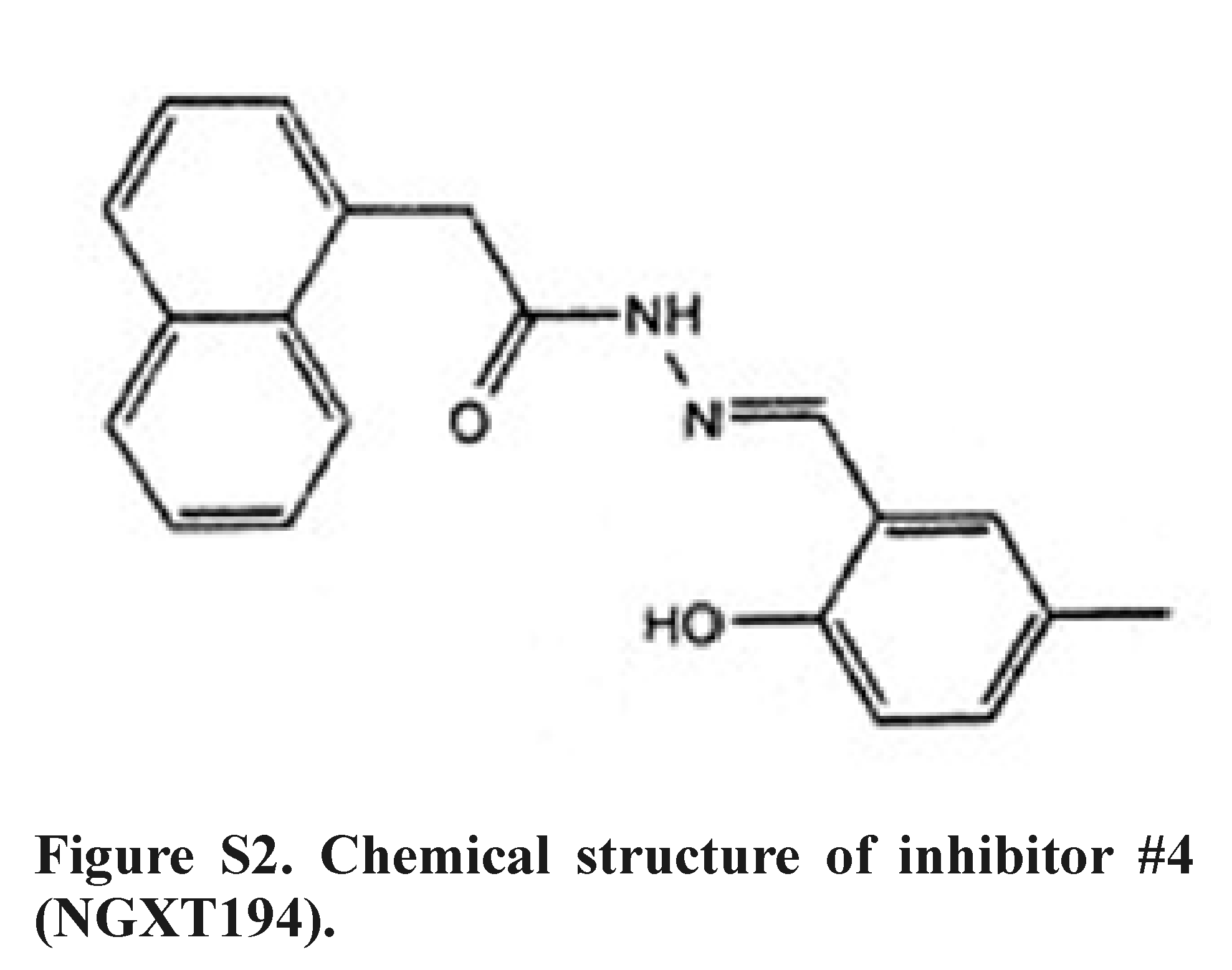

Supplement: S2 Fig — (TIF) [file pone.0115832.s002.tif]

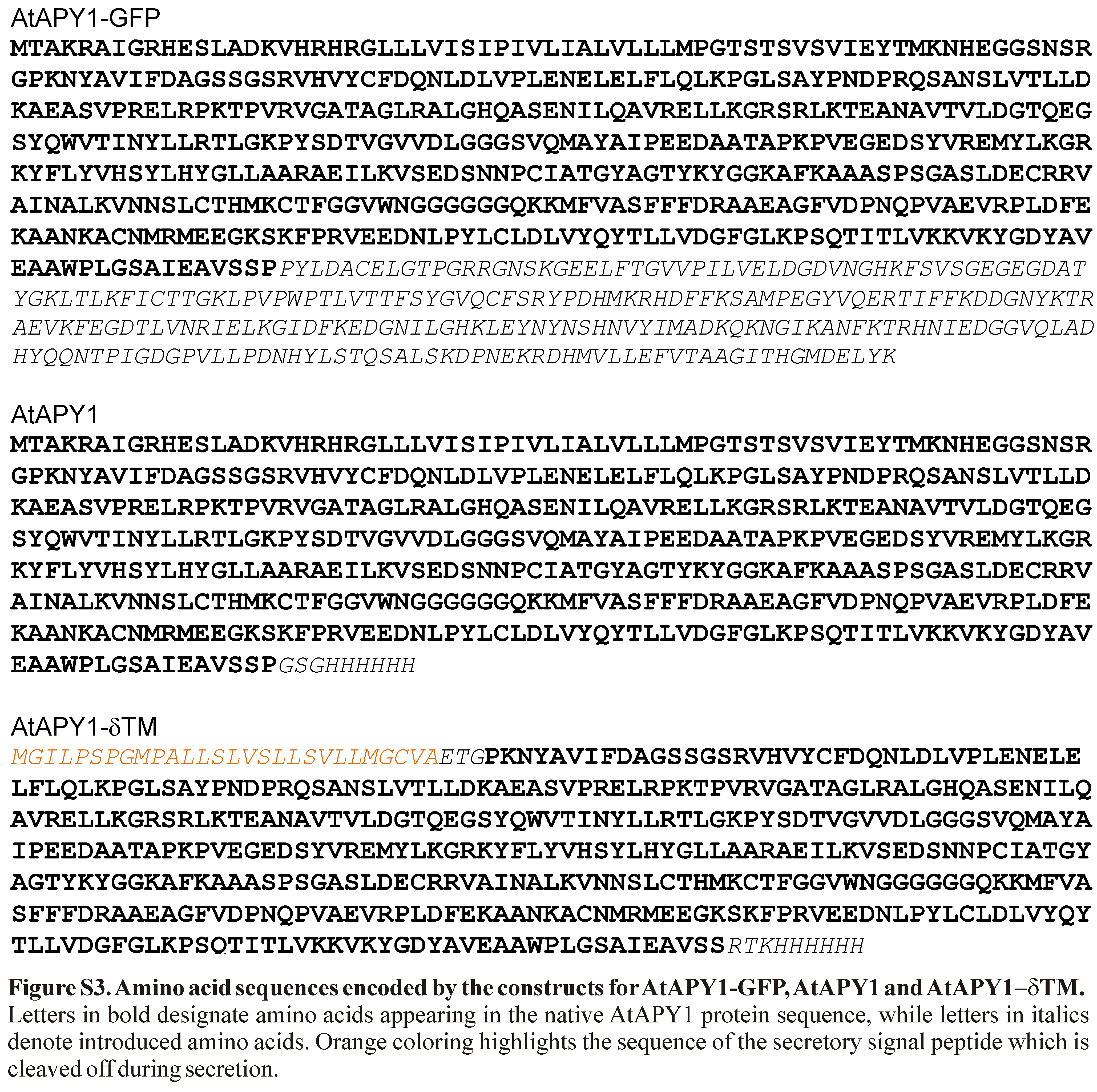

Supplement: S3 Fig — Letters in bold designate amino acids appearing in the native AtAPY1 protein sequence, while letters in italics denote introduced amino acids. Orange coloring highlights the sequence of the secretory signal peptide which is cleaved off during secretion. (TIF) [file pone.0115832.s003.tif]

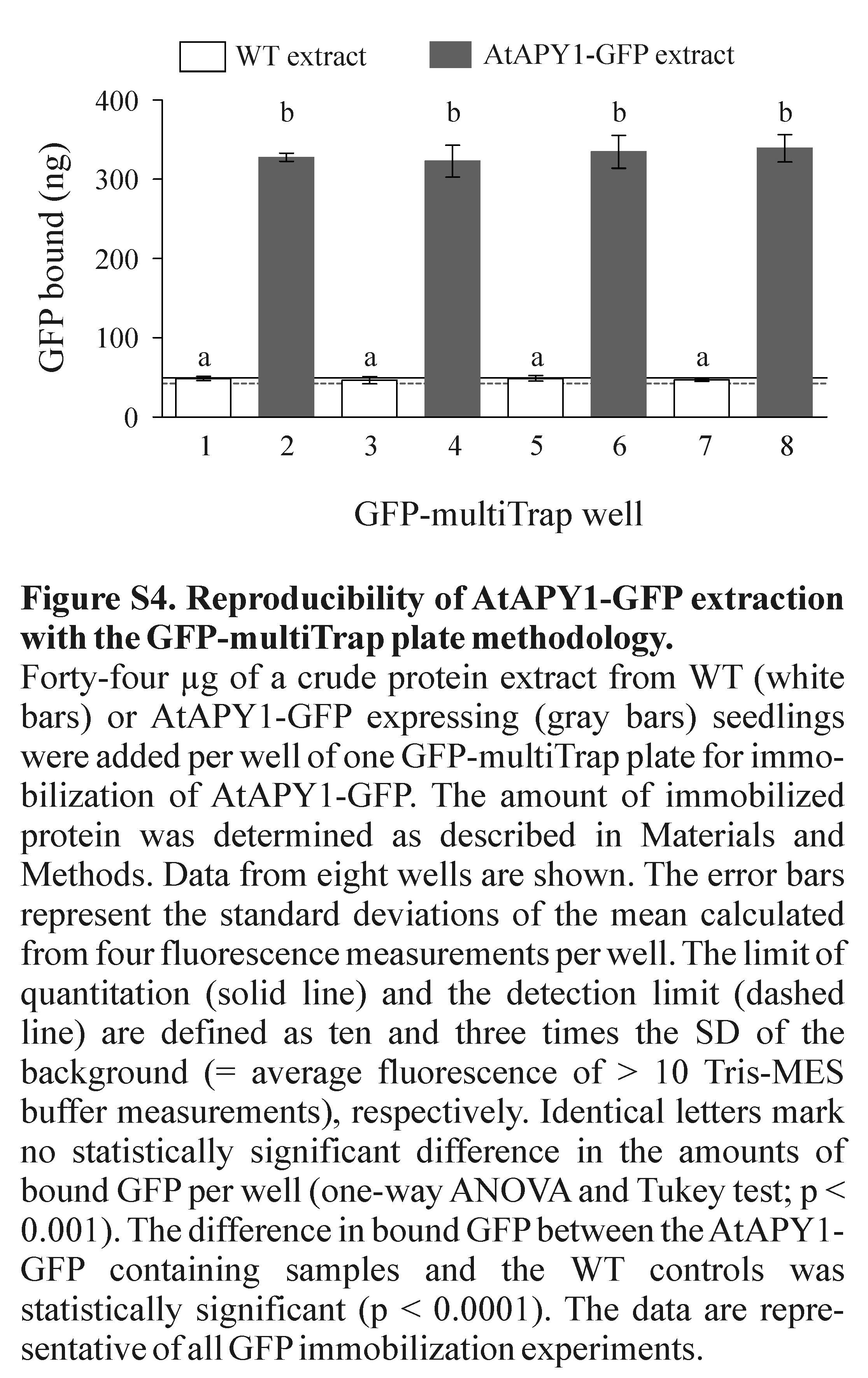

Supplement: S4 Fig — Forty-four μg of a crude protein extract from WT (white bars) or AtAPY1-GFP expressing (gray bars) seedlings were added per well of one GFP-multiTrap plate for immobilization of AtAPY1-GFP. The amount of immobilized protein was determined as described in Materials and Methods. Data from eight wells are shown. The error bars represent the standard deviations of the mean calculated from four fluorescence measurements per well. The limit of quantitation (solid line) and the detection limit (dashed line) are defined as ten and three times the SD of the background (= average fluorescence of > 10 Tris-MES buffer measurements), respectively. Identical letters mark no statistically significant difference in the amounts of bound GFP per well (one-way ANOVA and Tukey test; p < 0.001). The difference in bound GFP between the AtAPY1-GFP containing samples and the WT controls was statistically significant (p < 0.0001). The data are representative of all GFP immobilization experiments. (TIF) [file pone.0115832.s004.tif]

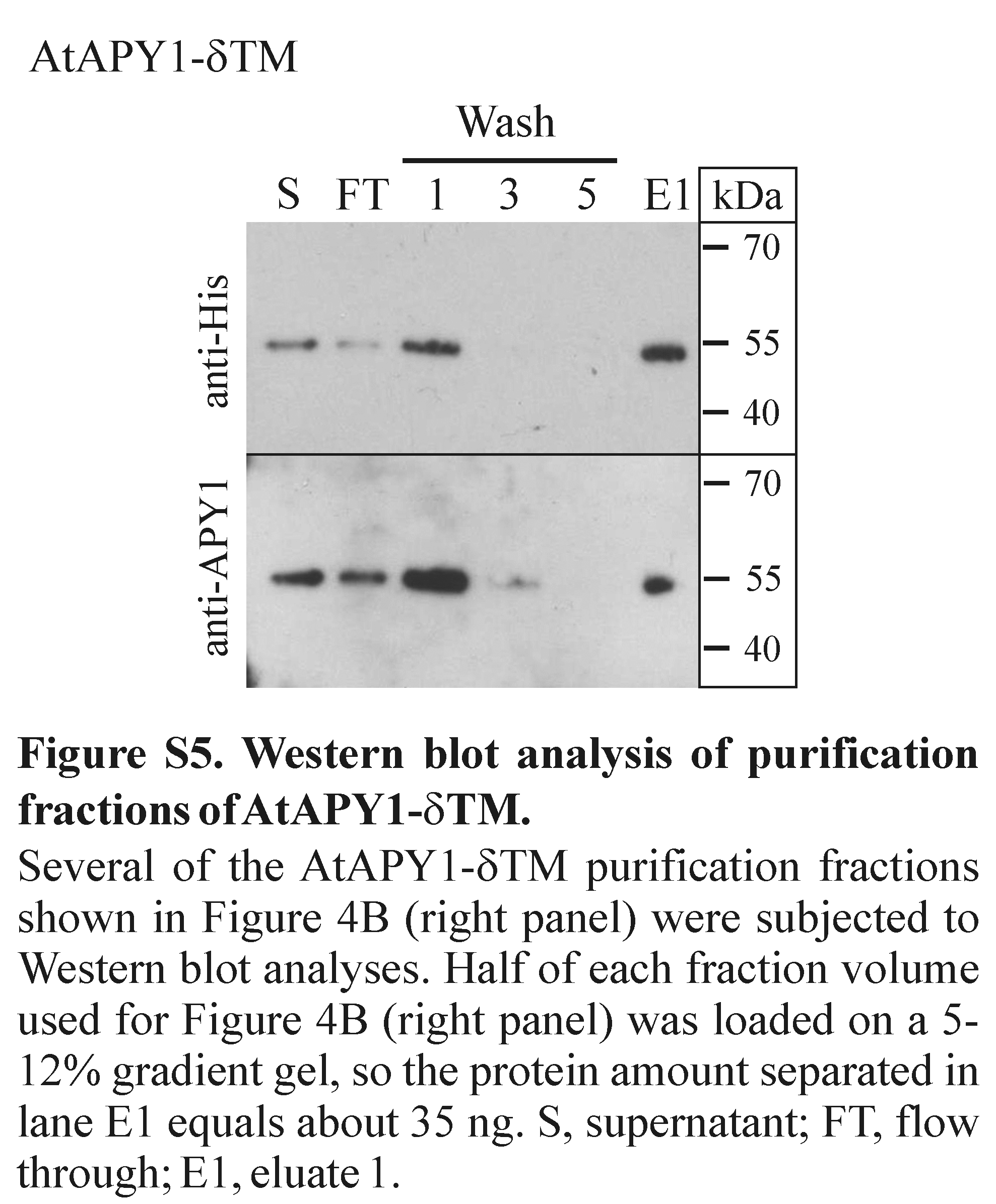

Supplement: S5 Fig — Several of the AtAPY1-δTM purification fractions shown in Fig. 4B (right panel) were subjected to Western blot analyses. Half of each fraction volume used for Fig. 4B (right panel) was loaded on a 5–12% gradient gel, so the protein amount separated in lane E1 equals about 35 ng. S, supernatant; FT, flow through; E1, eluate 1. (TIF) [file pone.0115832.s005.tif]

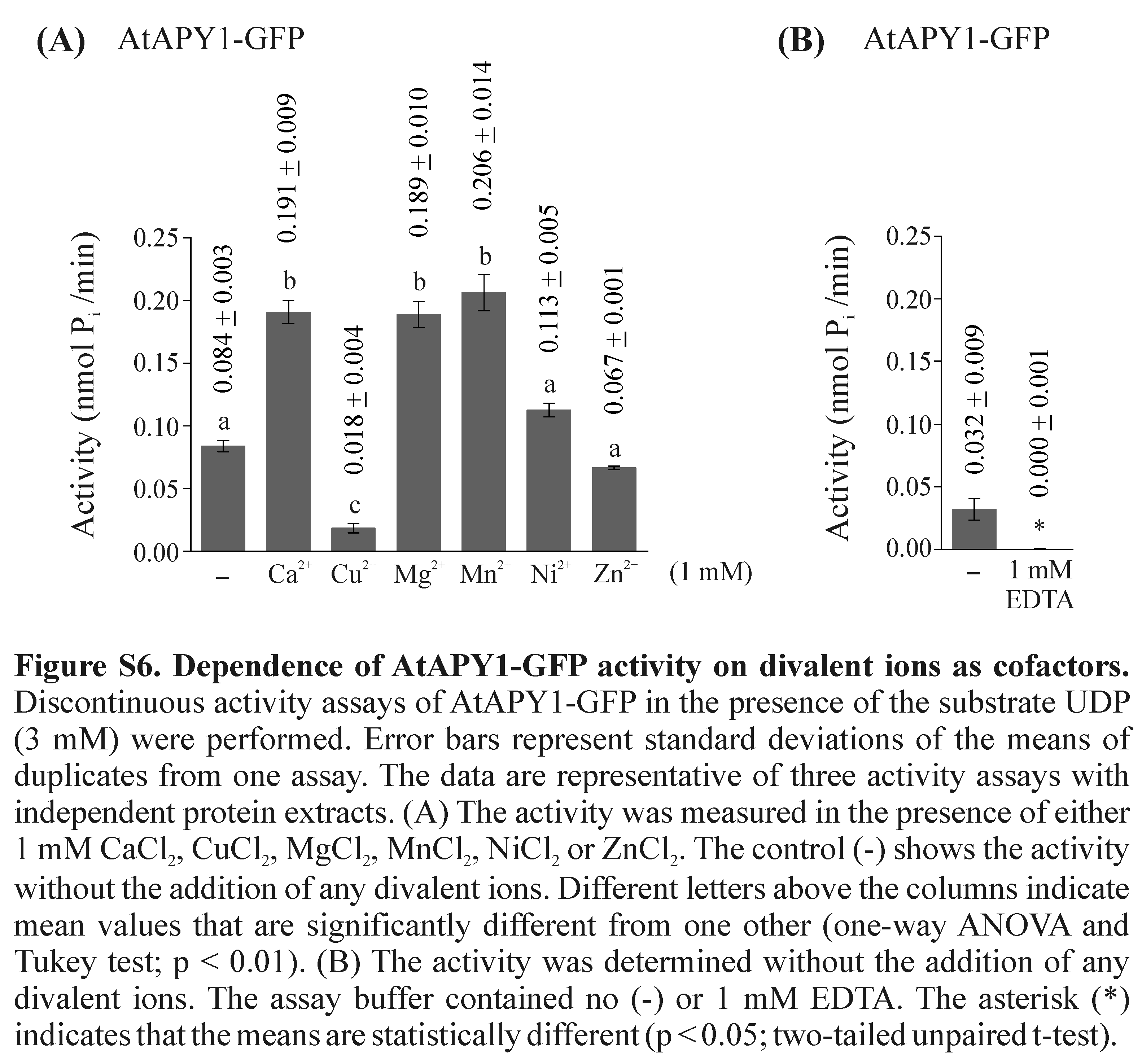

Supplement: S6 Fig — Discontinuous activity assays of AtAPY1-GFP in the presence of the substrate UDP (3 mM) were performed. Error bars represent standard deviations of the means of duplicates from one assay. The data are representative of three activity assays with independent protein extracts. (A) The activity was measured in the presence of either 1 mM CaCl2, CuCl2, MgCl2, MnCl2, NiCl2 or ZnCl2. The control (-) shows the activity without the addition of any divalent ions. Different letters above the columns indicate mean values that are significantly different from one other (one-way ANOVA and Tukey test; p < 0.01). (B) The activity was determined without the addition of any divalent ions. The assay buffer contained no (-) or 1 mM EDTA. The asterisk (*) indicates that the means are statistically different (p < 0.05; two-tailed unpaired t-test). (TIF) [file pone.0115832.s006.tif]

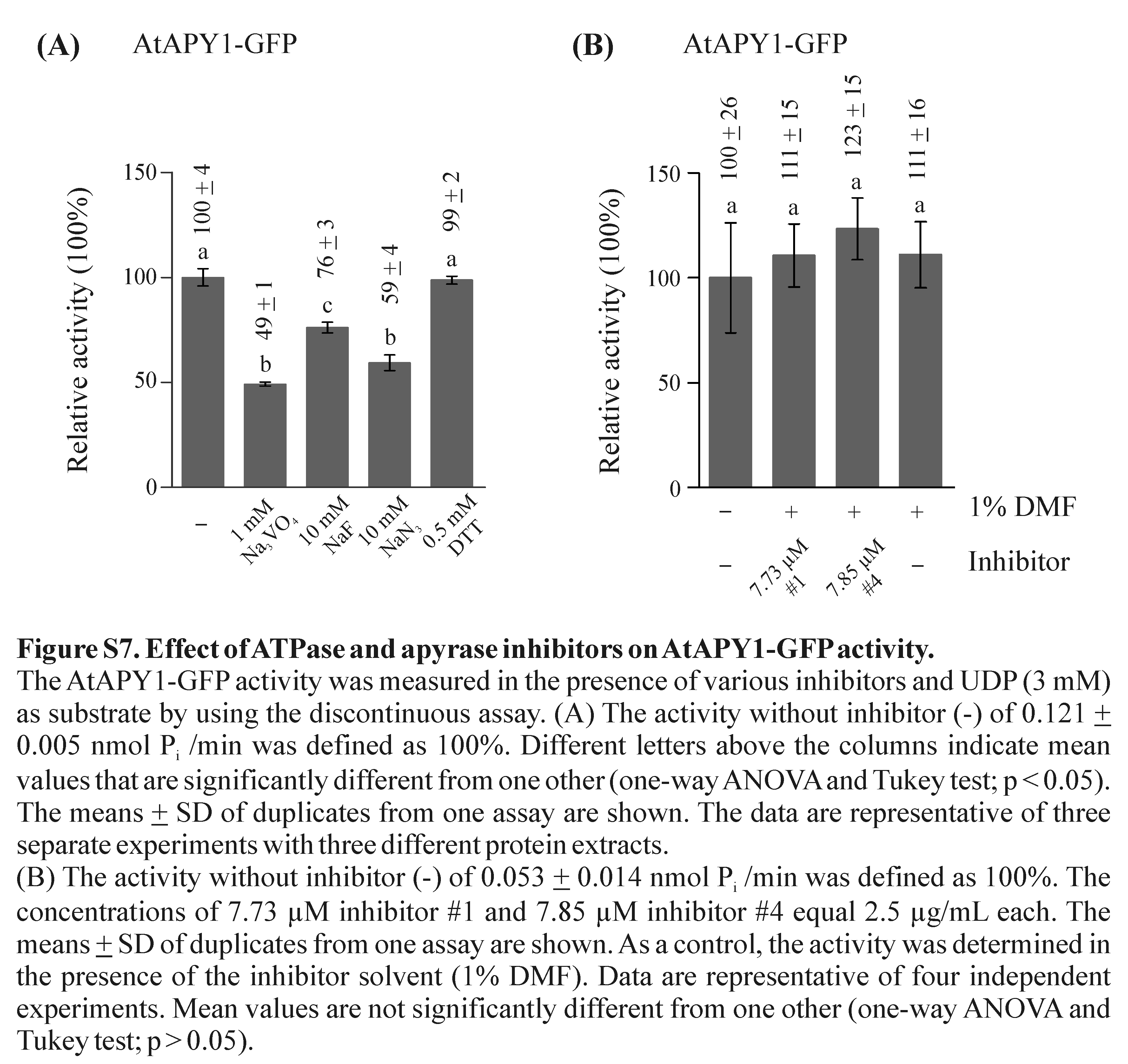

Supplement: S7 Fig — The AtAPY1-GFP activity was measured in the presence of various inhibitors and UDP (3 mM) as substrate by using the discontinuous assay. (A) The activity without inhibitor (-) of 0.121 + 0.005 nmol Pi /min was defined as 100%. Different letters above the columns indicate mean values that are significantly different from one other (one-way ANOVA and Tukey test; p < 0.05). The means + SD of duplicates from one assay are shown. The data are representative of three separate experiments with three different protein extracts. (B) The activity without inhibitor (-) of 0.053 + 0.014 nmol Pi /min was defined as 100%. The concentrations of 7.73 μM inhibitor #1 and 7.85 μM inhibitor #4 equal 2.5 μg/mL each. The means + SD of duplicates from one assay are shown. As a control, the activity was determined in the presence of the inhibitor solvent (1% DMF). Data are representative of four independent experiments. Mean values are not significantly different from one other (one-way ANOVA and Tukey test; p > 0.05). (TIF) [file pone.0115832.s007.tif]

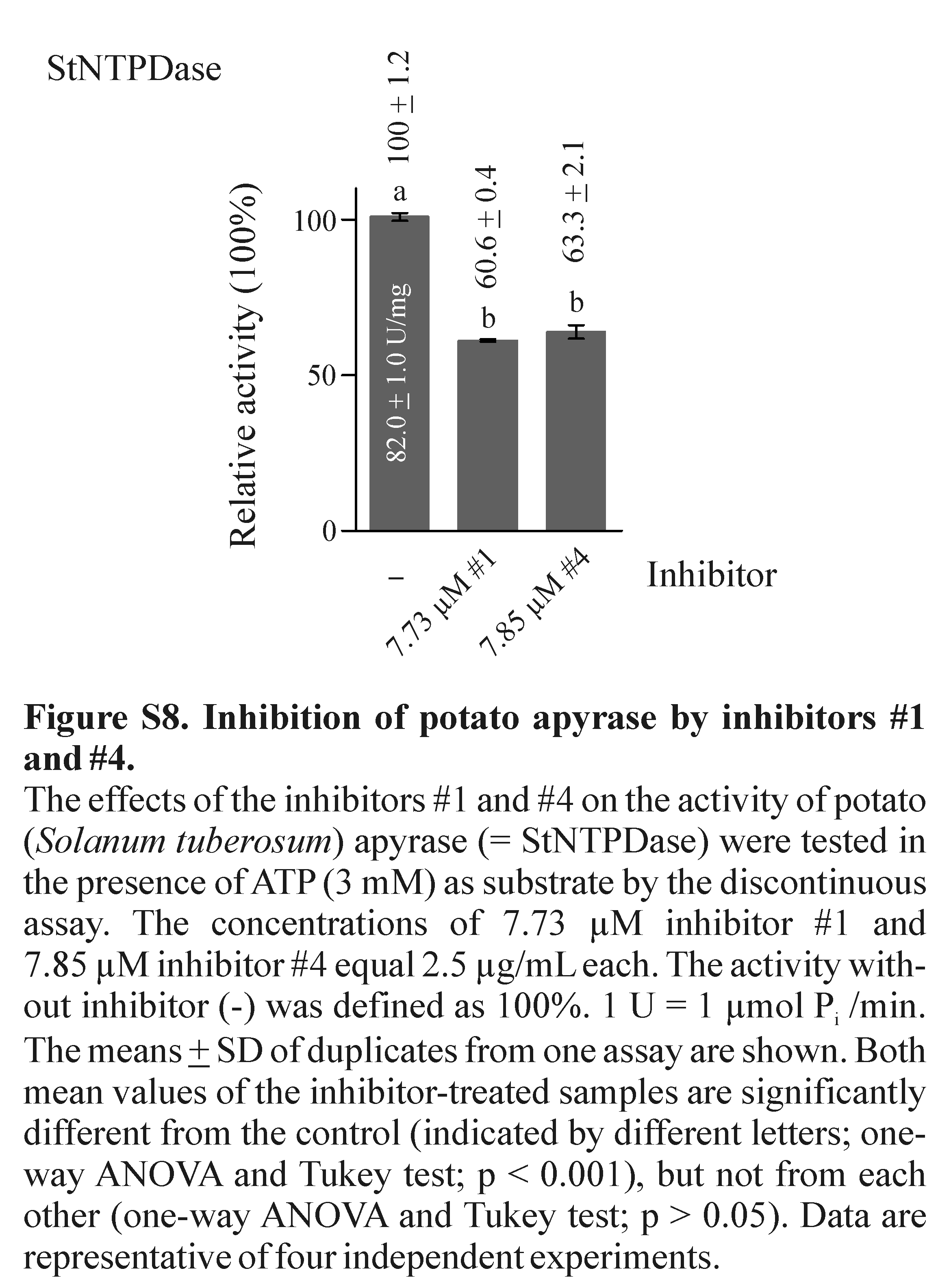

Supplement: S8 Fig — The effects of the inhibitors #1 and #4 on the activity of potato (Solanum tuberosum) apyrase (= StNTPDase) were tested in the presence of ATP (3 mM) as substrate by the discontinuous assay. The concentrations of 7.73 μM inhibitor #1 and 7.85 μM inhibitor #4 equal 2.5 μg/mL each. The activity without inhibitor (-) was defined as 100%. 1 U = 1 μmol Pi /min. The means + SD of duplicates from one assay are shown. Both mean values of the inhibitor-treated samples are significantly different from the control (indicated by different letters; one-way ANOVA and Tukey test; p < 0.001), but not from each other (one-way ANOVA and Tukey test; p > 0.05). Data are representative of four independent experiments. (TIF) [file pone.0115832.s008.tif]

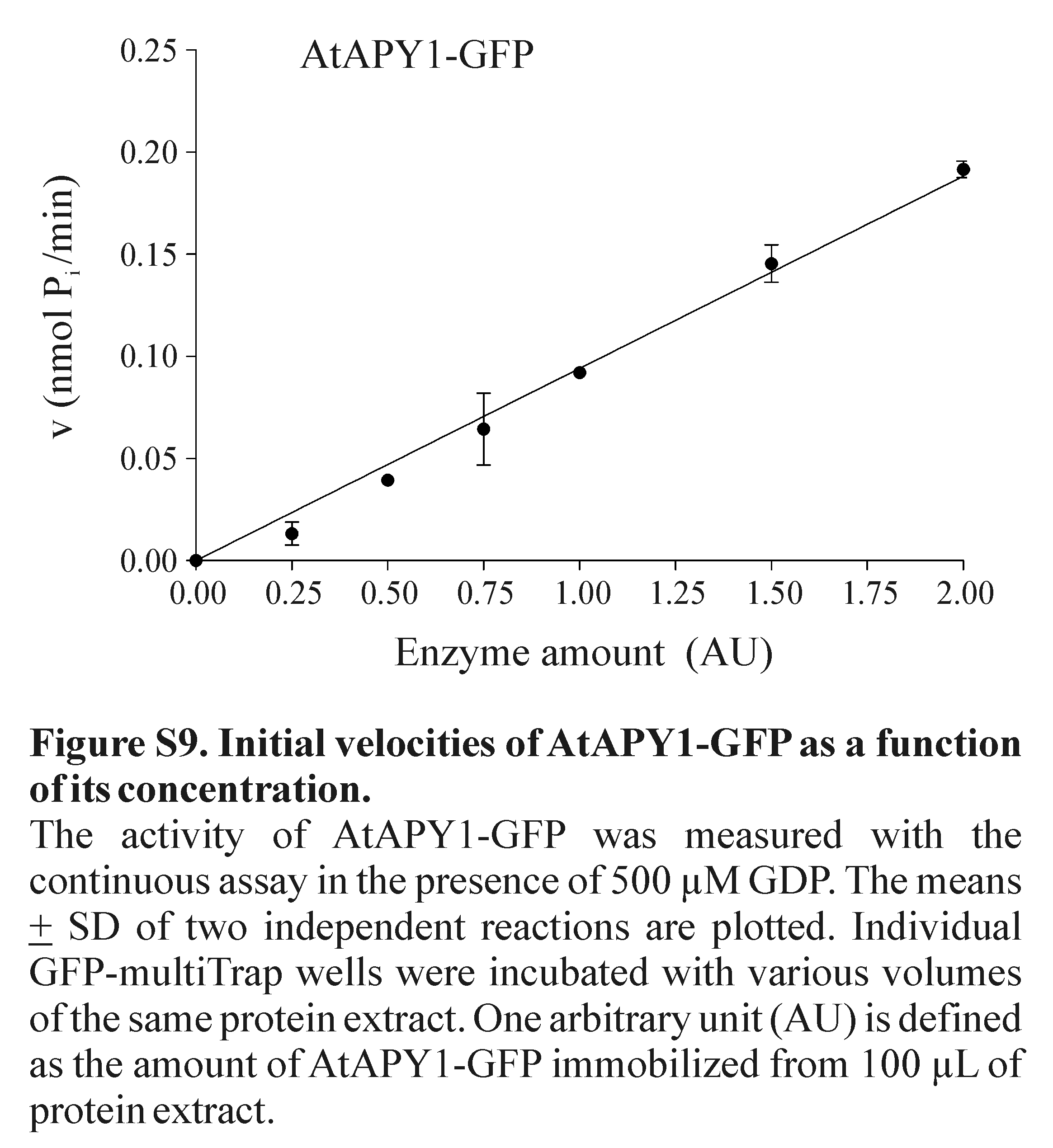

Supplement: S9 Fig — The activity of AtAPY1-GFP was measured with the continuous assay in the presence of 500 μM GDP. The means + SD of two independent reactions are plotted. Individual GFP-multiTrap wells were incubated with various volumes of the same protein extract. One arbitrary unit (AU) is defined as the amount of AtAPY1-GFP immobilized from 100 μL of protein extract. (TIF) [file pone.0115832.s009.tif]

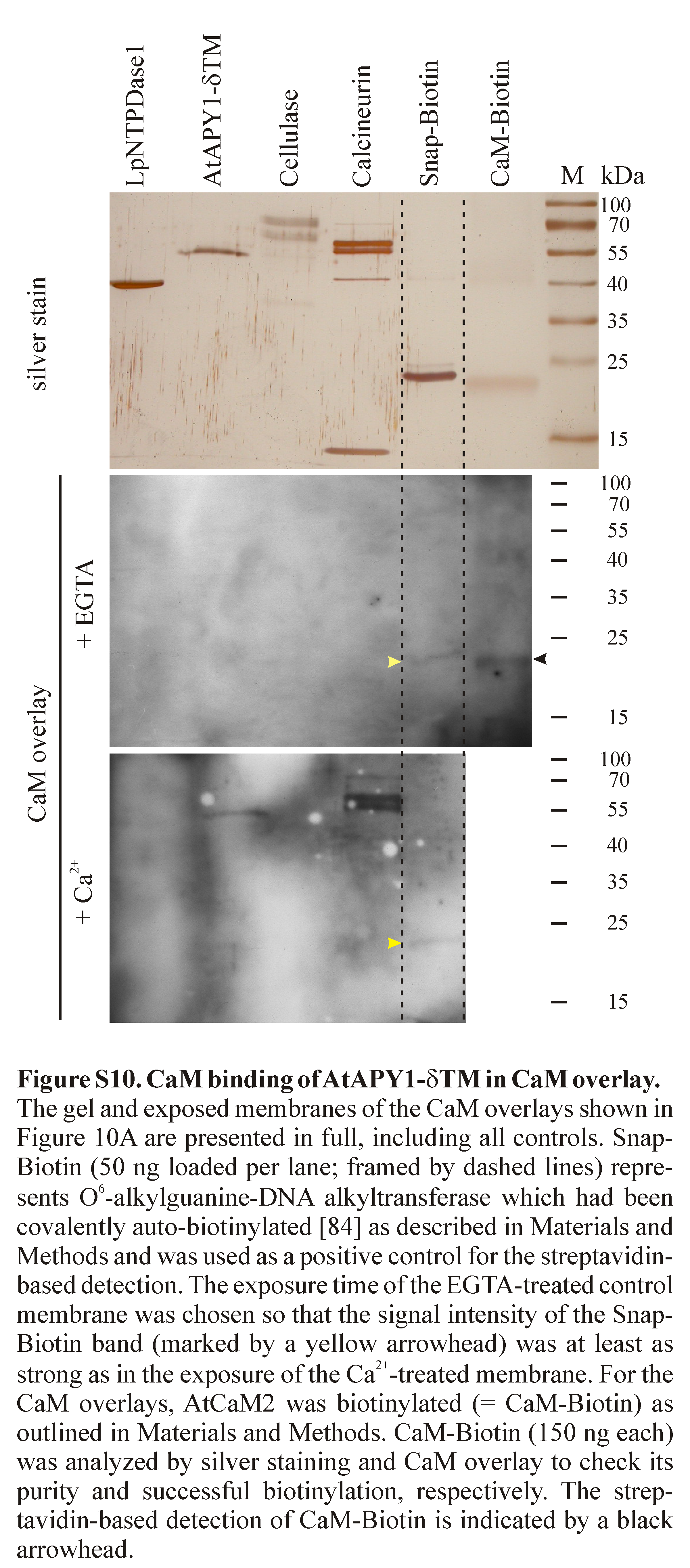

Supplement: S10 Fig — The gel and exposed membranes of the CaM overlays shown in Fig. 10A are presented in full, including all controls. Snap-Biotin (50 ng loaded per lane; framed by dashed lines) represents O6-alkylguanine-DNA alkyltransferase which had been covalently auto-biotinylated [84] as described in Materials and Methods and was used as a positive control for the streptavidin-based detection. The exposure time of the EGTA-treated control membrane was chosen so that the signal intensity of the Snap-Biotin band (marked by a yellow arrowhead) was at least as strong as in the exposure of the Ca2+-treated membrane. For the CaM overlays, AtCaM2 was biotinylated (= CaM-Biotin) as outlined in Materials and Methods. CaM-Biotin (150 ng each) was analyzed by silver staining and CaM overlay to check its purity and successful biotinylation, respectively. The streptavidin-based detection of CaM-Biotin is indicated by a black arrowhead. (TIF) [file pone.0115832.s010.tif]

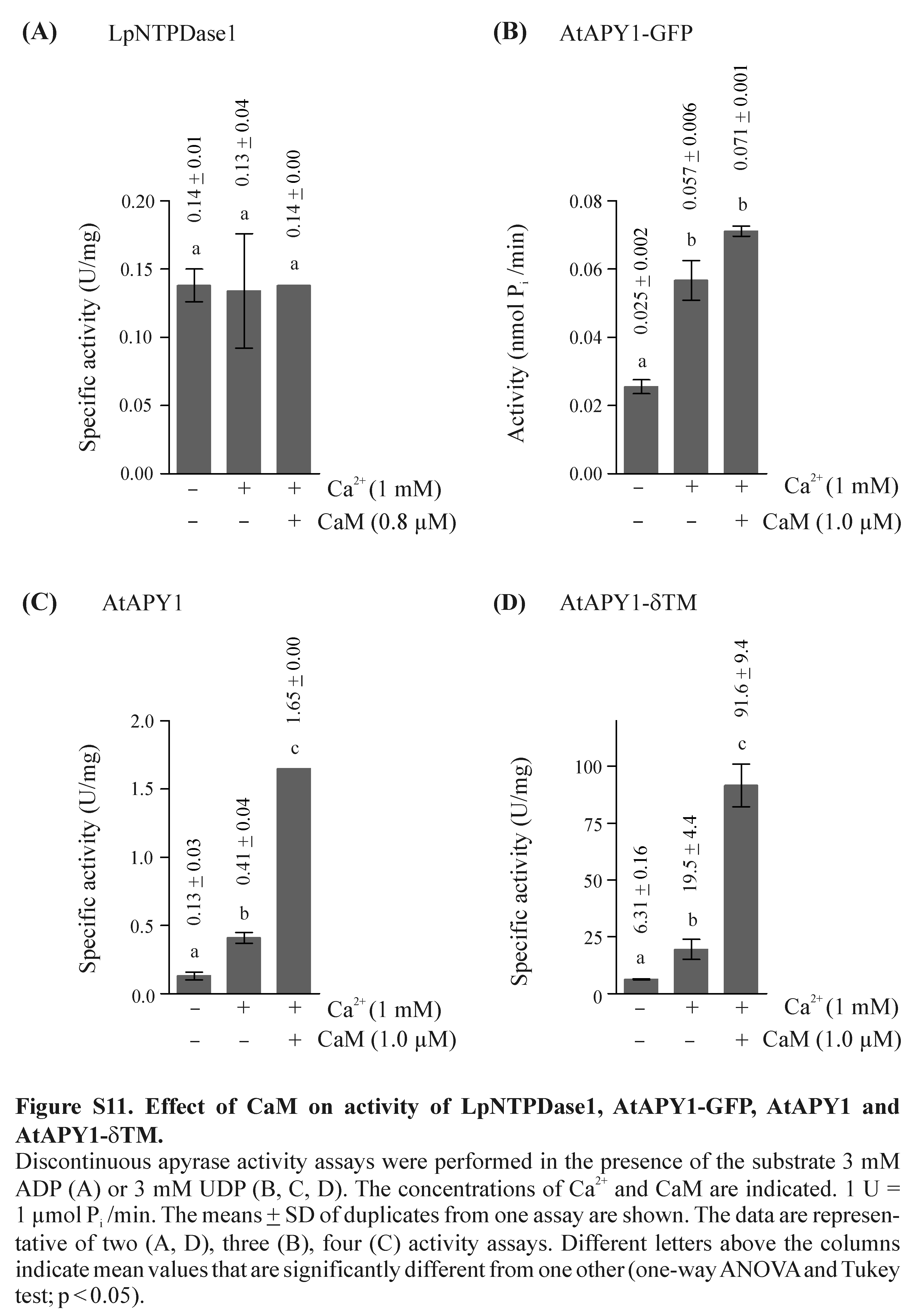

Supplement: S11 Fig — Discontinuous apyrase activity assays were performed in the presence of the substrate 3 mM ADP (A) or 3 mM UDP (B, C, D). The concentrations of Ca2+ and CaM are indicated. 1 U = 1 μmol Pi /min. The means + SD of duplicates from one assay are shown. The data are representative of two (A, D), three (B), four (C) activity assays. Different letters above the columns indicate mean values that are significantly different from one other (one-way ANOVA and Tukey test; p < 0.05). (TIF) [file pone.0115832.s011.tif]

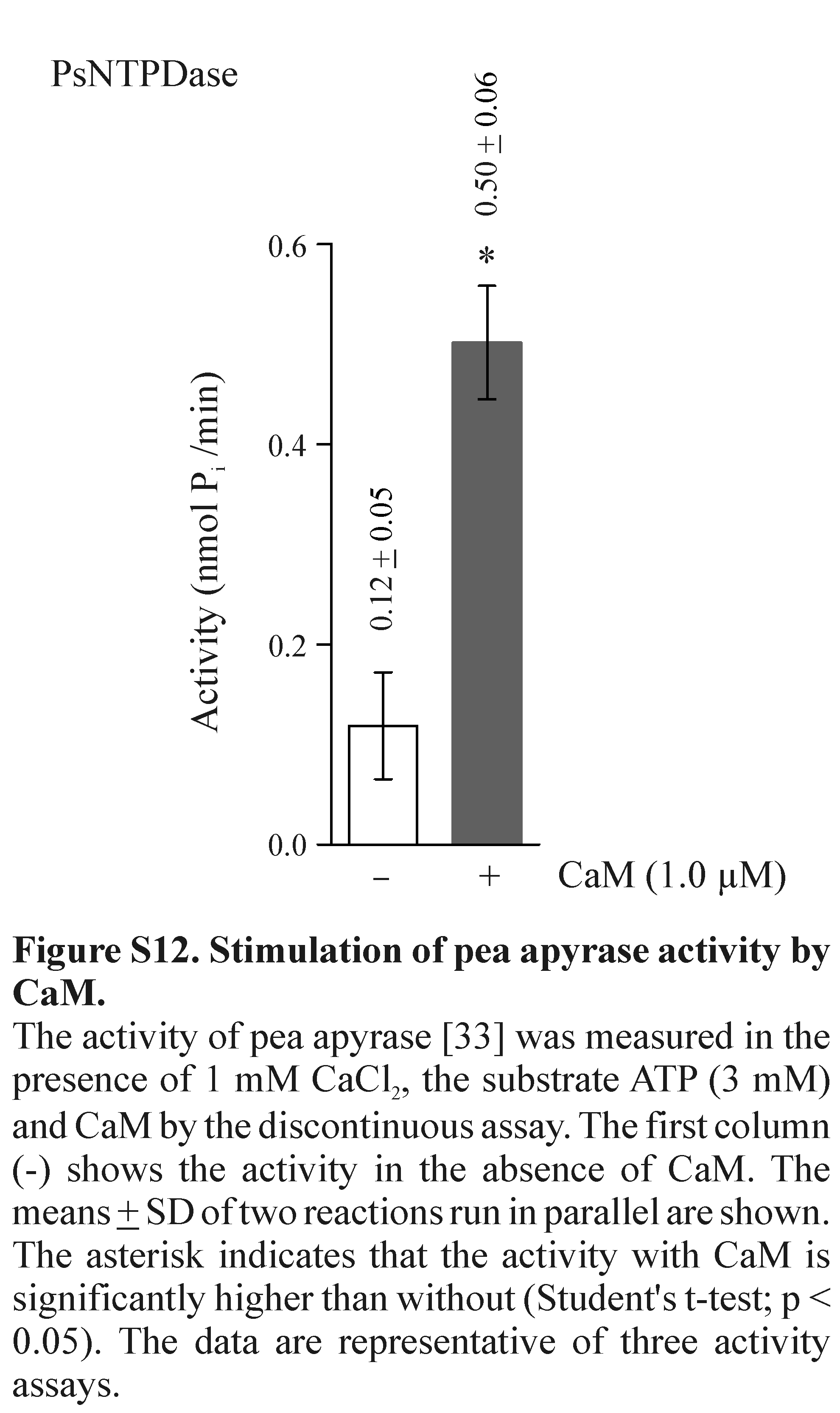

Supplement: S12 Fig — The activity of pea apyrase [33] was measured in the presence of 1 mM CaCl2, the substrate ATP (3 mM) and CaM by the discontinuous assay. The first column (-) shows the activity in the absence of CaM. The means + SD of two reactions run in parallel are shown. The asterisk indicates that the activity with CaM is significantly higher than without (Student’s t-test; p < 0.05). The data are representative of three activity assays. (TIF) [file pone.0115832.s012.tif]
